# The influence of time and visualization on neurofeedback-guided parietal alpha downregulation and sense of presence in virtual reality

**DOI:** 10.3389/fnins.2025.1476264

**Published:** 2025-02-12

**Authors:** Loic Botrel, Alex Kreilinger, Mathias Müller, Maria Pfeiffer, Vincent Scheu, Nico Vowinkel, Roland Zechner, Ivo Käthner, Andrea Kübler

**Affiliations:** ^1^Julius Maximilian University of Würzburg, Würzburg, Germany; ^2^Brain Products GmbH, Gilching, Germany; ^3^VTplus GmbH, Würzburg, Bavaria, Germany

**Keywords:** neurofeedback, high density EEG, portable EEG, inverse connectivity, virtual reality

## Abstract

In an EEG-based near real-time neurofeedback (NF) study in two parts using high immersive virtual reality (VR) we successfully trained healthy participants to downregulate their parietal alpha power, a neurophysiological correlate previously associated with enhanced sense of presence. The first part included *n* = 10 participants equipped with 128 and 64 channels gel-based active EEG electrodes in 10 sessions using standard bar feedback presented on a computer monitor. Nine participants were better than random at the 10th session and four improved over time. For the second part we reduced the electrode subset to 9 sponge-based active channels (2 frontal, 7 parietal around Pz) and a portable amplifier. Participants (*n* = 10) were trained each session within VR using bar feedback projected on a wall in the first 5 sessions and then controlling the flow of a water fountain. Participants were able to significantly downregulate their parietal alpha power after 5 sessions and learning occurred at the group level, with 7 participants showing both improvement over time and ability to modulate. However, these results were only shown during the fountain feedback and both ability and learning were non-significant in the VR projector condition. Based on self-reports, after excluding participants performing movements and closing their eyes, no particular mental strategy, such as relaxation, breathing or mental calculus was identified to help with alpha modulation. The hypothesized behavioral effect on sense of presence was not found nor any neurophysiological changes in fronto-parietal connectivity. While NF did not improve the sense of presence, we succeeded in adapting real-time NF training for high immersive VR technology via seamlessly embedded feedback in the form of a water fountain. The study showcases that NF is possible with sponge electrodes and portable EEG that would prove convenient in end-user (at home) or clinical setup. The dataset is publicly available on Openneuro.org.

## Introduction

Immersive and interactive virtual reality (VR) has become widely available since 2013 when the first consumer grade head-mounted display (HMD) prototype destined for mass market was publicly announced at Consumer Electronics Show (CES). It enabled high immersive VR via tracking the users’ movements. Combined with broadly available 3D development tools, it is now possible to generate virtual environments with virtual agents that provide immersive experiences. While applications have initially been most popular for gaming, there are many promising applications of HMD devices in education, exercise and healthcare (for review, Hamad and Jia, 2022).

HMD-presented VR (HMD-VR) has been successful at creating immersive experiences (Slater and Sanchez-Vives, 2014) and eliciting heightened sense of presence in virtual environment (VE, Steuer, 1992). Participants experience a sense of “being there” in the VE (Steuer, 1992) in opposition to where the body currently is (Slater and Usoh 1993). Although often used synonymously, immersion and sense of presence can be distinguished as follows: immersion is quantifiable by objective measures and refers to the system’s ability to present an environment highly realistically, while blocking or replacing sensory inputs (Sanchez-Vives and Slater, 2005; Slater and Wilbur, 1997). In contrast, sense of presence is a subjective sensation of the participant experiencing something as really happening (for further disambiguation between immersion and sense of presence, see Slater, 1999). More immersive environments lead to higher sense of presence (Cummings and Bailenson, 2016; Yildirim et al., 2019).

Recent studies have investigated neural correlates of the sense of presence (Baumgartner et al., 2006). They subjected participants to two VR rollercoaster tracks, one very flat and the other labeled as “spectacular.” The most arousing track was associated with an increased sense of presence and an increased activity in the parietal cortex. Authors found a prefrontal-parietal top-down inhibition that was associated with decreased sense of presence. Baumgartner et al. (2008) replicated their study using functional magnetic resonance imaging (fMRI) and found in low presence participants a similar top-down inhibition pattern involving the right dorsolateral prefrontal cortex (dlPFC), and large areas covering the parietal cortex involving the superior parietal cortex (SPG), the precuneus (IPG), and the parietal cingular cortex (PCC). Kober and colleagues reported similar findings (Kober et al., 2012). They asked the participants to navigate through a maze, displayed either on a monitor or via an interactive VR wall. The interactive VR wall condition, that evoked a higher sense of presence, was associated with a decrease in power as compared to baseline indicating increased parietal activation. Decrease in time-related power (TRPD) were observed in the low alpha range (8-12 Hz) at the right parietal (Pz, P2 and P4) and right parieto-occipital (POz, PO2 and PO4) channels. Time-related power inhibition (TRPI) and TRPD express the power during a time window in percentage of a separate reference window such as a baseline recording. In contrast, event-related synchronization/desynchronization (ERS/ERD, Pfurtscheller, 1992) are measured in relation to time windows with individual baselines preceding their related event. The results also showed a stronger functional connectivity between frontal and parietal areas in the low presence group (i.e., using a monitor). The authors interpreted the increase in parietal activity as a result of adopting an egocentric, body-centered view evoked by the interactive VR wall.

On the basis of these results we were aiming at decreasing parietal alpha activity by means of neurofeedback (NF, for review see Enriquez-Geppert et al., 2017) to increase the sense of presence. NF allows for regulation of physiological activity that is usually not perceivable. Instrumental or operant learning (Miller, 1978; Skinner, 1963) is involved in acquiring such skill. The goal of gaining control of (neuro)physiological activity is to change the related behavior. For example to increase attention by increasing the amplitude of slow cortical potentials (Birbaumer et al., 1990). Neurofeedback learning leads to neuroplasticity and then presumably to behavioral change (Gruzelier, 2009). It has been used for various applications such as epileptic seizure reduction (Wyrwicka and Sterman, 1968), attention deficit hyperactivity syndrome in children (Arns et al., 2014), anxiety reduction (Stokes and Lappin, 2010), cognitive improvement (Dessy et al., 2018), migraine pain reduction (Roy et al., 2020), or to improve cognitive performance after stroke (Kleih and Botrel, 2024). After about 60 years of existence, NF still faces concerns with respect to evidence of the causal relationship between the achieved neuromodulation and behavioral changes, and its specificity (Loriette et al., 2021; Thibault and Raz, 2017). One of the reasons of the lack of evidence may be in the tremendous heterogeneity of study protocols, and reports thereof, and sample sizes. To overcome these shortcomings in the future, guidelines have been provided (Rogala et al., 2016) and more recently a consensus has been co-signed by 84 researchers from 80 affiliations aimed at improving standards and reporting of NF studies (Ros et al., 2020). In both herein presented studies we strictly followed these recommendations and reported when criteria could not be reached.

Our two studies were motivated by two factors. Firstly, the potential effect on pain reduction associated with increased sense of presence caused by parietal alpha downregulation. It has been shown that using immersive VR can reduce pain in the context of severe burn injury treatments (Maani et al., 2011; Hoffman et al., 2011) and has been researched as an adjunct treatment for central neuropathic pain (Roy et al., 2020). Secondly, we were aiming at transposing NF training from a classical 2D presentation to 3D HMD-VR and to integrate the EEG electrodes and amplifier on the HMD. The 3D presentation should be as similar as possible to classic (bar) NF while at the same time be highly immersive. The classic bar feedback seemed essential, as it has been proven that it allows for gaining control over brain activity (Kober et al., 2015). There are several limitations associated with the use of HMD and EEG electrodes in one system. The electrode positions are limited adaptable and may cause pressure leading to discomfort after little time.

Provided previous research, these two studies presented here were an attempt to answer firstly, whether we could increase the sense of presence with NF. For this, Part 1 assessed whether it was possible to train a neurophysiological correlate of the sense of presence and how much training would be needed. The consecutive Part 2 adjusted and translated the NF training of Part 1 fully inside HMD-VR.

The study in two parts described here were conducted within a project that aimed at reducing chronic pain via the use of VR and BCI, and to provide an easy-to-apply VR-BCI-NF treatment for reducing chronic pain. In a first step – presented in this paper – the sense of presence, which is negatively associated with pain perception (Hoffman et al., 2011), should be increased with NF training by increasing the neuronal correlate associated with the sense of presence. Part 1 concentrated on testing if it was possible to regulate parietal alpha activity and to explore its effects on the sense of presence. Part 2 emphasized on adapting the NF procedure for a more practical approach and therefore the number of electrodes were reduced. Since we were aiming at demonstrating that NF effects both, the neurophysiological and behavioral responses, and NF was required to be practical in clinical and home daily life set-ups, an NF scenario was integrated in VR and transfer sessions without NF were conducted in VR to evaluate the effect of NF training on the sense of presence when no feedback is provided.

To encourage reproducibility and further research, dataset is publicly available for part 1 10.18112/openneuro.ds005846.v1.0.0 and part 2 10.18112/openneuro.ds005878.v1.0.0.

### Hypotheses

We pre-registered the methods, dependent variables and hypotheses on the platform aspredicted.org (Wharton Lab, University of Pennsylvania, USA).^1^ For this manuscript we improved the original formulation into the following hypotheses:

- Parietal alpha power is reduced during NF training after 10 sessions.
- Parietal alpha power does not differ whether feedback is presented or hidden (transfer).
- Parietal alpha power does not differ whether full feedback or positive-only feedback is provided.
- Baseline parietal alpha activity remains stable across sessions.
- NF-based modulation of parietal alpha power is possible with a reduced subset of electrodes (offline comparison).
- NF Training has no long-term effect on sense of presence.
- Increase in fronto-parietal connectivity dlPFC (Broadman area A46) PCC (BA23) and PPC (BA5) as a result of training.
- Participants’ reported fatigue before recording baseline correlates with baseline alpha power

## Part 1

### Materials and methods

#### Participants

Part 1 was conducted in 2021. Participants (*n* = 10, mean age 28.3, SD = 9.5, 5 female, 50% psychology students) attended 10 neurofeedback training sessions within a time frame of 3 weeks at the institute of Psychology Würzburg, University of Würzburg (Germany). Ethical approval was granted by the Ethical Review Board of the Institute of Psychology at the Faculty for Human Sciences, University of Würzburg (GZEK 2021-36). Participants were rewarded with 10 Euros per hour, but with a minimum of 40 Euros guaranteed. For Psychology students course credits were provided alternatively.

**Figure 1:**
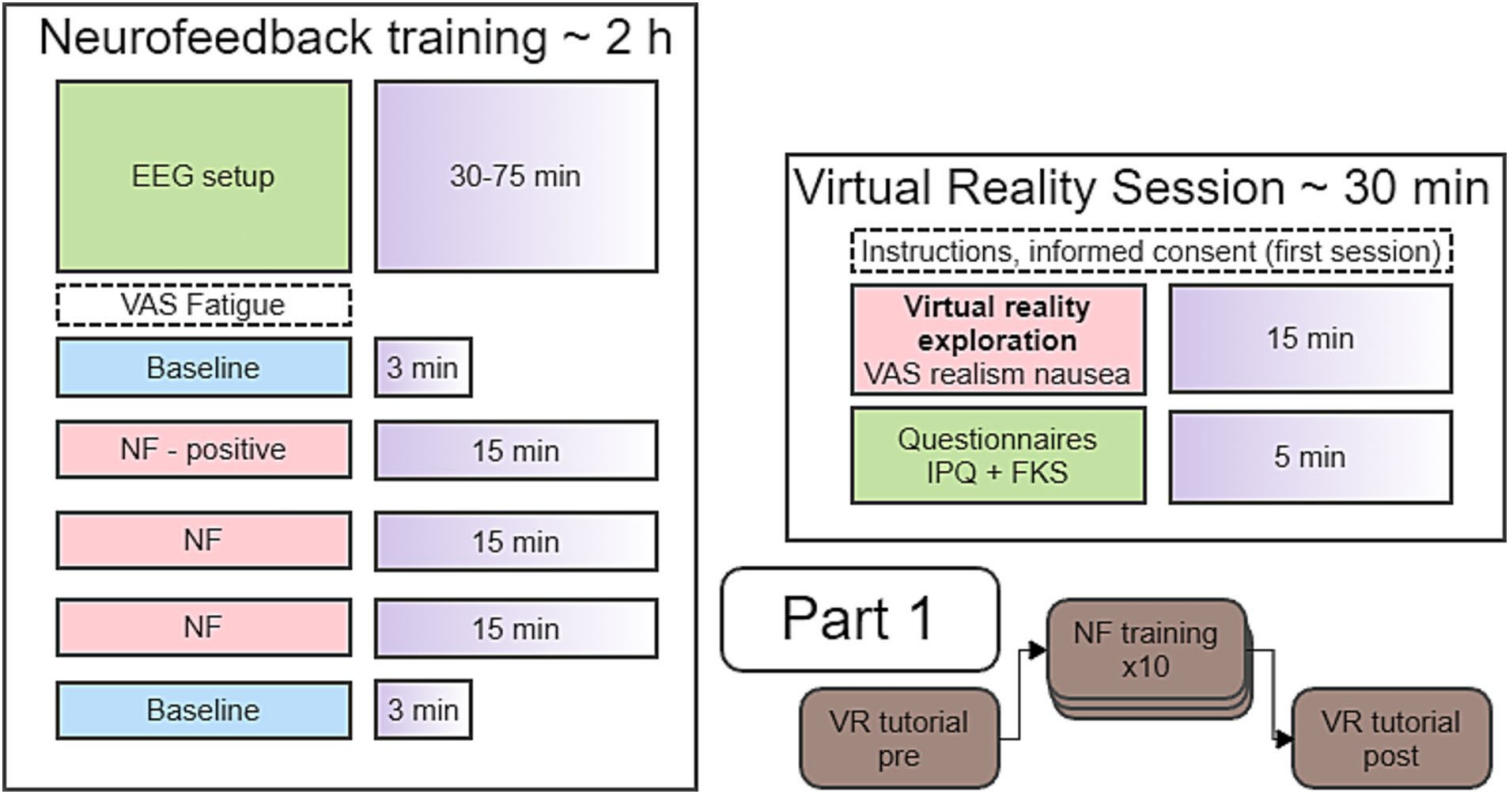
Timeline of the sessions in Part 1.

Participants took part in an immersive VR “walk around” session (“pre”) to assess the sense of presence. After completion, participants continued with the first of 10 NF sessions. NF sessions were repeated on separate days within 3 weeks. Also, to avoid fatigue, the second (“post”) VR “walk around” was done at least one day after the 10^th^ NF session (see Figure 1 for study timeline).

#### Virtual reality setup

**Figure 2:**
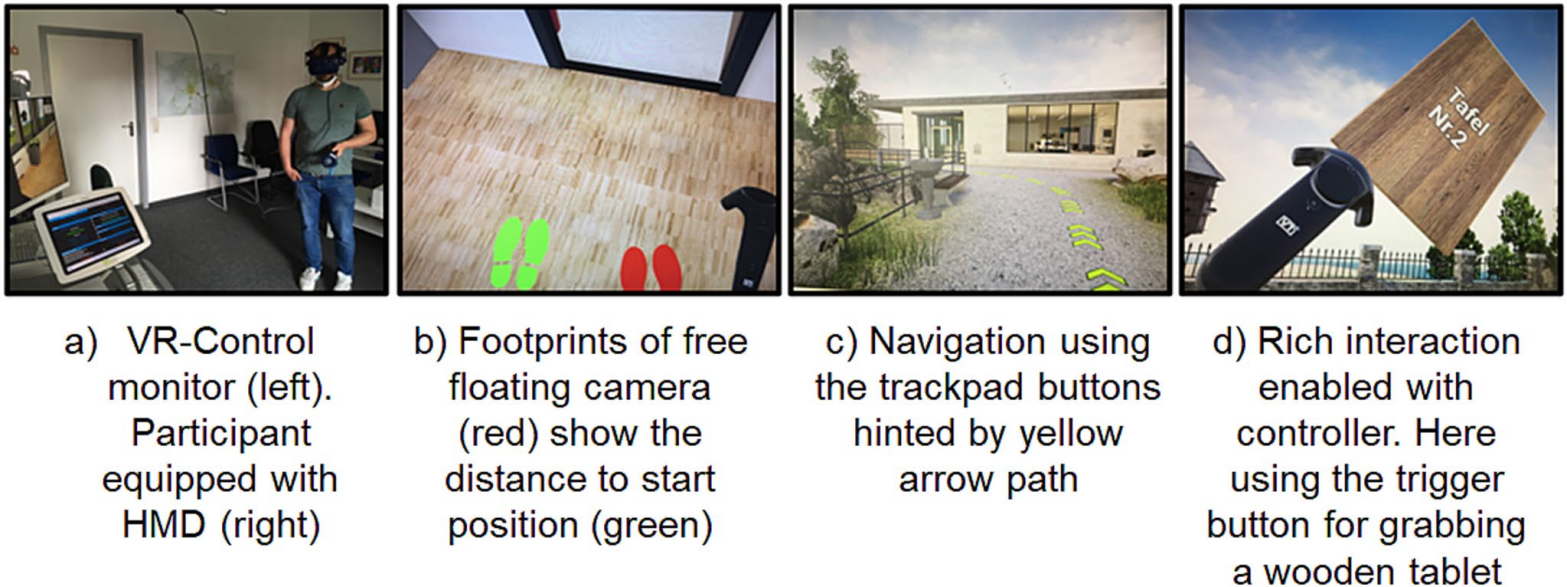
Showcase of the virtual reality device and participant **(A)** and its in-game features including spatial cues [footprints **(B)**, and directional arrows **(C)**] and augmented interactions with the motion-tracked controller **(D)**. Note that the floor-projected footprints **(B)** guide the participants to their initial physical position (outside VR) to reduce the occurrence of collisions in VR and outside VR. When triggered, the task is to physically move to make the location of the observer’s point of view (red footprints) overlap with the virtual solid avatar (green footprints).

The VTplus ExpoCart3 VR system was provided by VTplus (GmbH) and simulated a realistic scenery using the HTC Vive Pro HMD, which tracks head and controller position in the room and translates it in the VE. The system had a dedicated computer unit, a monitor that mirrored the view of the HMD display and a touchpad control panel for the experimenter. From the control panel, the experimenter could start the VR scenario, which consisted of different steps of VTplus’ VR tutorial that taught participants to move in the VR via the joystick controller, grab objects using controller tracking and button press to complete simple interaction tasks (see Figure 2). The tutorial designed for participants with no previous VR experience also featured countermeasures for VR sickness: movement initiated by the directional buttons was not smooth but consisted of incremental shifts of about half a meter each. After each movement update from the joystick, there was a transient directional reduction of the field of view for reducing cybersickness as recommended by Fernandes and Feiner (2016). The tracking answered naturally to head movements, yet, using natural walking movement to move to target location was discouraged as with 7 m^2^ the space for real walking was much smaller than that presented in VR space. The user started the scenario anchored in an invisible avatar with the point of view (camera) matching with the head position tracked by the HMD sensors. Tracked movements would accordingly translate the camera in VR in an unobstructed fashion (i.e., clipping through obstacles). Every movement on the controller moved both the anchor and the attached camera. To evolve in VR, the invisible avatar was constrained by the game engine, i.e., gravity to stick to the ground and collisions with walls. As time passes in VR, users become unaware of their position in the real world and the camera can venture too far from the anchor, participants then find themselves unable to navigate between obstacles, in particular though doors. As a solution, before navigation events, experimenters triggered an event to re-anchor the camera. A projection on the floor represented as footprints, showed the respective location of both camera (red) and anchor (green). Participants were asked to make both footprints coincide, which had the effect of relocating and reorienting them to their start position.

In Part 1 the VR system and NF systems were located in different rooms of the same building. NF was not provided in VR space.

#### Neurofeedback setup

**Figure 3:**
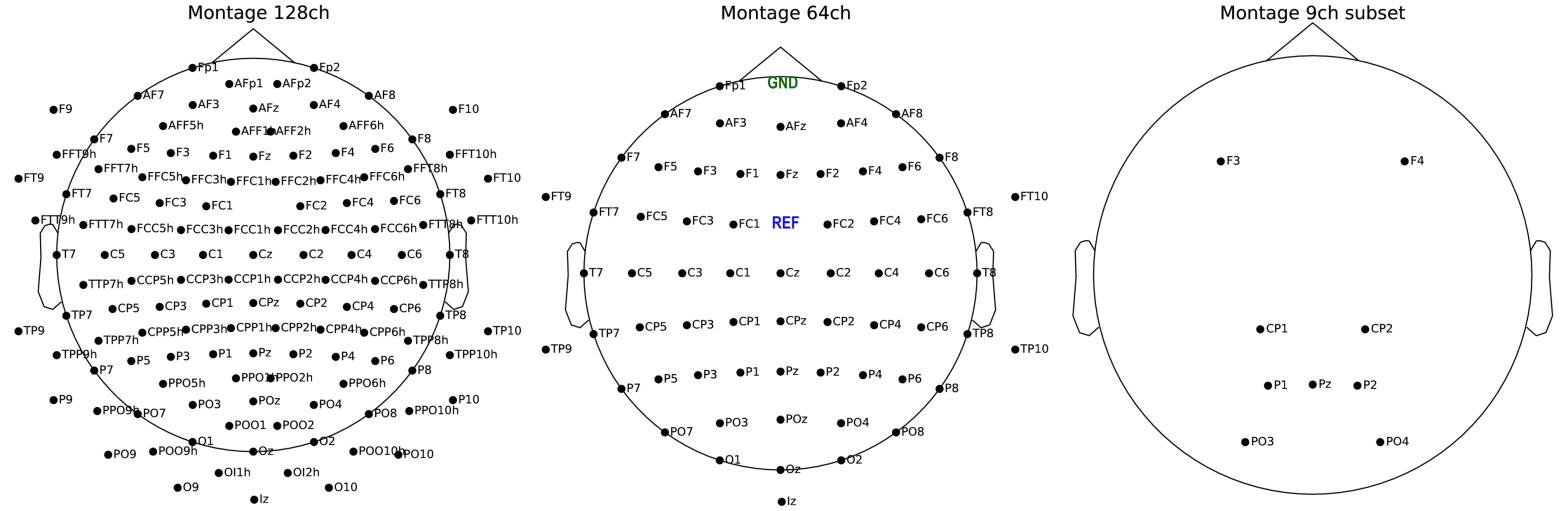
Montage of 128 channels active electrode system (left), 64 channels (center) and the reduced subset with 9 channels (right). Ground (GND) and reference (REF) for all three montage are visible on the center plot.

EEG signal was acquired by a 128 active channels actiCAP slim system. To reduce time-consuming preparation times, we used only 64 channels between sessions 2 to 9. The EEG was digitized by four BrainAmp amplifiers (Brain Products GmbH, Gilching, Germany). Electrodes were labeled following the 5–5 nomenclature (Oostenveld and Praamstra, 2001), after which we used FCz as reference channel and FPz for ground. The signal was digitized at a 500 Hz frequency expressed in microvolt [μV]. Participants sat on a chair with armrests in a sound-insulated EEG cabin and faced a 19-inch monitor at about 1.3 m distance. For visualizing montages see Figure 3.

#### Behavioural measures

##### Igroup presence questionnaire (IPQ)

To assess the sense of presence we used the Igroup presence questionnaire (IPQ, Schubert et al., 2001). The IPQ was specifically created for assessing presence in VE and was originally designed for German speakers. It was developed after factorial analysis which resulted in 14 items. Every item required to select an integer ranging from -3 to +3 complemented with text such as “not at all” or “fully disagree” on the negative and “very much” or “fully agree” on the positive end. The first item “general presence” directly referred to the sense of presence asked in German “In the computer generated world I had a sense of being there” (after Slater and Usoh, 1993). While we were specifically interested in this specific item, the questionnaire had three additional subscales: (1) Spatial presence (SP, *n* = 5 items): the sense of being physically present in the VE; (2) involvement (INV, *n* = 4 items): measuring the attention devoted to the VE; (3) Realism (REAL, *n* = 4 items): measuring the subjective experience of realism. The first item and the three subscales are independent factors. Hence, we did not aggregate them.

##### Short flow scale (flow-Kurzskala, FKS)

The Short Flow Scale (FKS) measures flow experience. It is an extension of the Rheinberg’s short “questionnaire about the experience of activities”, itself based on the work of Thiel & Kopf (1989) about the experience and frequency of flow. Using the experience sampling method, the FKS has 16 items that yield 3 factors called process (German: “automatisierter Verlauf”), flow (German: “Absorbiertheit”) and concern (German: “Besorgnis”).

#### VAS

Visual analog scales were provided to assess the level of realism, unease and nausea at the end of the VR exploration. They were presented during VR in the form of visual slides appearing on a 17-inch info panel at head level. The initial slide explained that the answers had to be provided orally and instructed to say “next,” after which the experimenter manually switched to the next slide. The first slide asked the participant to rate their experience by a number ranging from 0 “not real at all” to 10 “very real.” The second one asked about uneasiness between “no uneasiness” to “very strong uneasiness.” The third slide asked about nausea between “no nausea” to “very strong nausea.”

#### Study timeline

All NF training sessions had an identical timeline. Each started with a baseline run, a positive-only NF run, two full feedback NF runs and a post baseline run (see Figure 1).

Informed consent documents were provided digitally via email prior to the session and also available on paper format. It comprised the full timeline of Part 1 and all instructions about the VR sessions and NF training. Participants were blinded in two aspects: Firstly, they were informed that the targeted feature to modulate was parietal alpha, but we intentionally hid the direction of the relevant change in power (i.e., desynchronization). Secondly, we did not inform them about the behavioral target of the study (i.e., increase the sense of presence). The questionnaires used to measure presence were labeled as “measuring the quality of the VR experience.” If the experimenter was asked about the purpose of the study, the answer was that we were aiming at studying the modulation of parietal alpha since it was associated with cybersickness. Blinding and deceiving were revealed to the participant by the experimenter immediately at the end of the study. Instructions were provided by the experimenter orally, but also directly on the monitor prior to the baseline and NF runs. Due to the lack of respective knowledge, the experimenter was careful not to provide participants any suggestion about what mental strategy should be used to generate a change of parietal alpha activity and not to indicate the required direction. Such neutral instructions were, e.g., the alpha power had to be “steered,” “modulated” or “oriented,” in opposition to words such as “reduced” or “inhibited.”

##### NF sessions

Sessions 1 and 10 lasted longer due to the extra preparing time needed for *n* = 128 gel-based EEG channels. Participants were then instructed orally to keep their neck, jaw, face and overall head muscles relaxed and avoid strong eye movements as they generate massive artifacts in the EEG. To illustrate these instructions, participants were shown their live EEG activity filtered between 1 to 40 Hz. We asked them to move the head and neck, clench their teeth, blink their eyes repeatedly 3 or 4 times, close their eyes for about 10 s, glance strongly right and left, frown or tap their feet nervously. This step allowed the experimenter to check the EEG signal for potentially inadequately prepared “loose” channels by looking at how much the amplitude drifted and how long it took to readjust.

In two 90 s trials in one run, the baseline was recorded during which participants were asked to watch the animation of a 3D sphere made of white dots on a black background slowly rotating; trials were separated by a 15 s break. After calibration of the system that determined the average and range of the online signal, there were two online NF runs that lasted about 15 min. The first run with reduced expressivity had positive only feedback (i.e., from the origin, the bar moved only upward). The second run had full range feedback (the bar moved also down from the origin, indicating that the system detected a modulation in the wrong direction). Runs comprised 30 trials, split in 3 blocks with a 30 s break in between. Every 10^th^ trial was a “test trial” with no NF provided. We instructed participants to “modulate their brain activity” as in those runs with NF. Before initiating each run, there were short instructions displayed on screen, “lower the red bar that represents artifact,” a depiction of the cues for trials “move the bar up” or “move the bar up (no feedback)” and an example image of the feedback.

**Figure 4:**
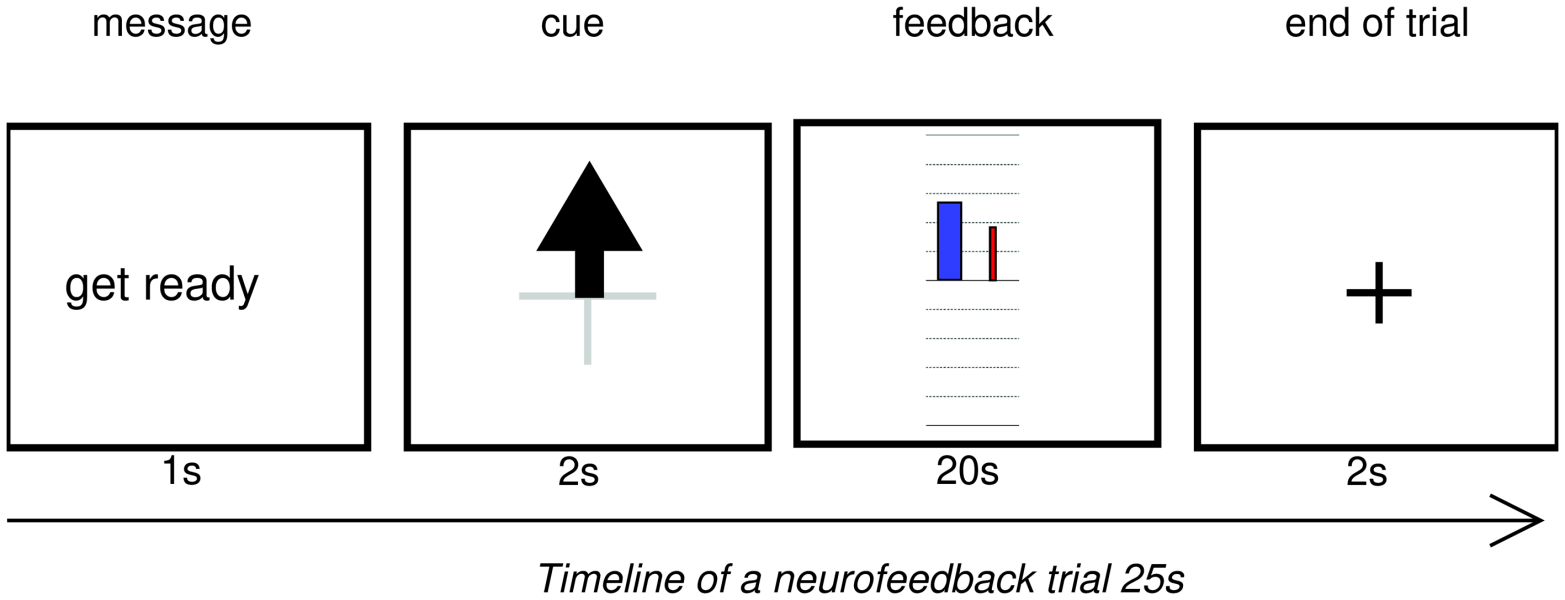
Timeline of a neurofeedback trial displayed on the experimenter’s monitor. Colors are inverted for readability. In experimental conditions, the text, arrow, grid and fixation cross were white on a black background.

Every trial started with a fixation cross for 2 s, followed by a “get ready” message for 1 s, then the (upward arrow) cue was displayed for 2 s. Then real-time NF was presented for 20 s. At the end of the trial, participants did not receive any reward, but a fixation cross appeared for 2 s (see Figure 4).

##### VR sessions

The VR session started with placing the HMD and ensuring it fitted properly by displaying an eye test chart on which participants were asked to read the letters and declare any anomaly. The HMD could accommodate the user wearing glasses. After placing the participant in the starting position, the virtual world was initialized, representing the yard of a one floor building. The participant performed a sequence of different tasks: (1) viewing in all directions; (2) walking to a target and back; (3) grabbing an object with the controller; (4) moving and bending toward a virtual water fountain; (5) opening doors and flipping a light switch; (6) collecting bank notes and depositing them inside a box. To ensure navigation was possible, the participant was re-anchored in VR several times per session. Once about 2 min after the beginning, before drinking from a water fountain (in VR), and circumstantially about 3 min later if unable to cross the first door. Recentering of the solid avatar was required to avoid collisions with the walls. Head tracking only offered a limited radius of movement, and the participant used the directional keypad from the hand controller for any substantial forward-backward movement and rotation (see Figure 2).

All instructions about the task were provided by a pre-recorded voice that either repeated the instruction on failure or validated the participant’s progress. There were extra directional arrows dynamically painted on the ground displayed to guide the participant to the next stop. The completion of the tasks in VR lasted 15 min. At the end, the experimenter removed the HMD headset. To avoid bias, the experimenter remained neutral, discrete and avoided unnecessary communication with the participant. The participant had a few seconds to re-habituate to the real world, after which the experimenter asked to answer the VAS scales and IPQ questionnaires at a nearby table. Then the session was finished, and the experimenter returned to a normal communicative state.

### Online feature extraction from EEG data

EEG signal was processed using Python 3.9 with packages numpy1.23.4, scipy1.10.0, pylsl1.16.0, and pyxdf1.16.3. For enabling EEG data analysis and processing we used MNE (Gramfort et al., 2013) with packages python-mne1.5.1, mne-connectivity0.5.0. As communication protocol between the amplifier and the computer we used Lab Streaming Layer (LSL).^2^ Event markers and internal communication between data processing module and display module also used LSL. Thus, we could use a third-party open-source software (LabRecorder^3^) to record both EEG and marker streams in the same file.

During online treatment of EEG data, efficiency optimizations were made to keep data processing time below 100 ms. In MNE the object containing data (i.e., “raw”) was initialized once then duplicated without EEG data but all information about channel position required for spatial filter computation. New EEG signals from the amplifier were then attached in real time to duplicated objects in a cost-effective manner. MNE source code was altered, specifically, we used current source density (CSD) to refine EEG channel signal to their reference-free Laplacian transformations (Kayser and Tenke, 2006; Perrin et al., 1989). The code from current source density (CSD) was split in two parts. A first part to determine the model based on baseline data on all 128 or 64 channels, that had an identical montage, and a second part that quickly applied the transformation online. For bandpass and notch filtering of signal epochs, we substituted MNE functions by Butterworth, forward and backwards scipy equivalent (i.e., “filtfilt”).

#### Alpha power extraction from baseline

The EEG signal acquired during baseline was bandpass filtered between 1 to 40 Hz, using acausal Butterworth filters of order 16 and 4, respectively. Artifact subspace reconstruction (ASR, SCM method, Euclidian, package meegkit0.1.3) was used to correct for eye blink artifacts in the signal (Mullen et al., 2015). It was then split in 2 s epochs, corresponding to the length of the online NF buffer. After ASR treatment, the artifact rejection was strict: epochs exceeding 100 μV amplitude on any channel were discarded. From the artifact free epochs, CSD spatial filtering was applied on the data. Power spectrum density (PSD) was extracted using Welch’s method with nfft = 512. The 3 Hz wide window between 6.8 Hz to 12.7 Hz with the maximum PSD average, determined the alpha peak frequency range. PSD values were converted to dB using 20*log(x) and averaged across frequencies. Since we were using power values, the inaccurate dB formula was corrected during offline analysis to 10*log(x). Also, for readers’ convenience we refer to dB transformed PSD as “PSD.” The reference for normalizing PSD into *Z*-score was recomputed every session using the first baseline. The post-session baseline was not used. Each baseline lasted 180 s split into *n* = 90 epochs of 2 s.

#### Alpha power extraction during trials

The NF in online trials was updated every 100 ms, during which the software processed 2 s epochs of signal from 128 channels. Single epochs were bandpass filtered. Then, ASR and CSD models from the baseline were applied on the epoch before extracting PSD from the determined peak window. The mean baseline was subtracted from the extracted PSD and then divided by the standard deviation of the baseline to obtain a *Z*-score. This normalization expresses the power in standard deviations of the reference data (i.e., the baseline). An increase or decrease of power translated numerically with 0 indicating no difference, but also with a 95% theoretical probability that the online signal falls within the limits of −1.96 to 1.96 assuming it follows the same mean and distribution as the baseline. Such normalization combined with 10 overlapping updates per second allowed the feedback to fluctuate evenly and smoothly on the monitor without any manual intervention in setting up thresholds.

#### Feedback

**Figure 5:**
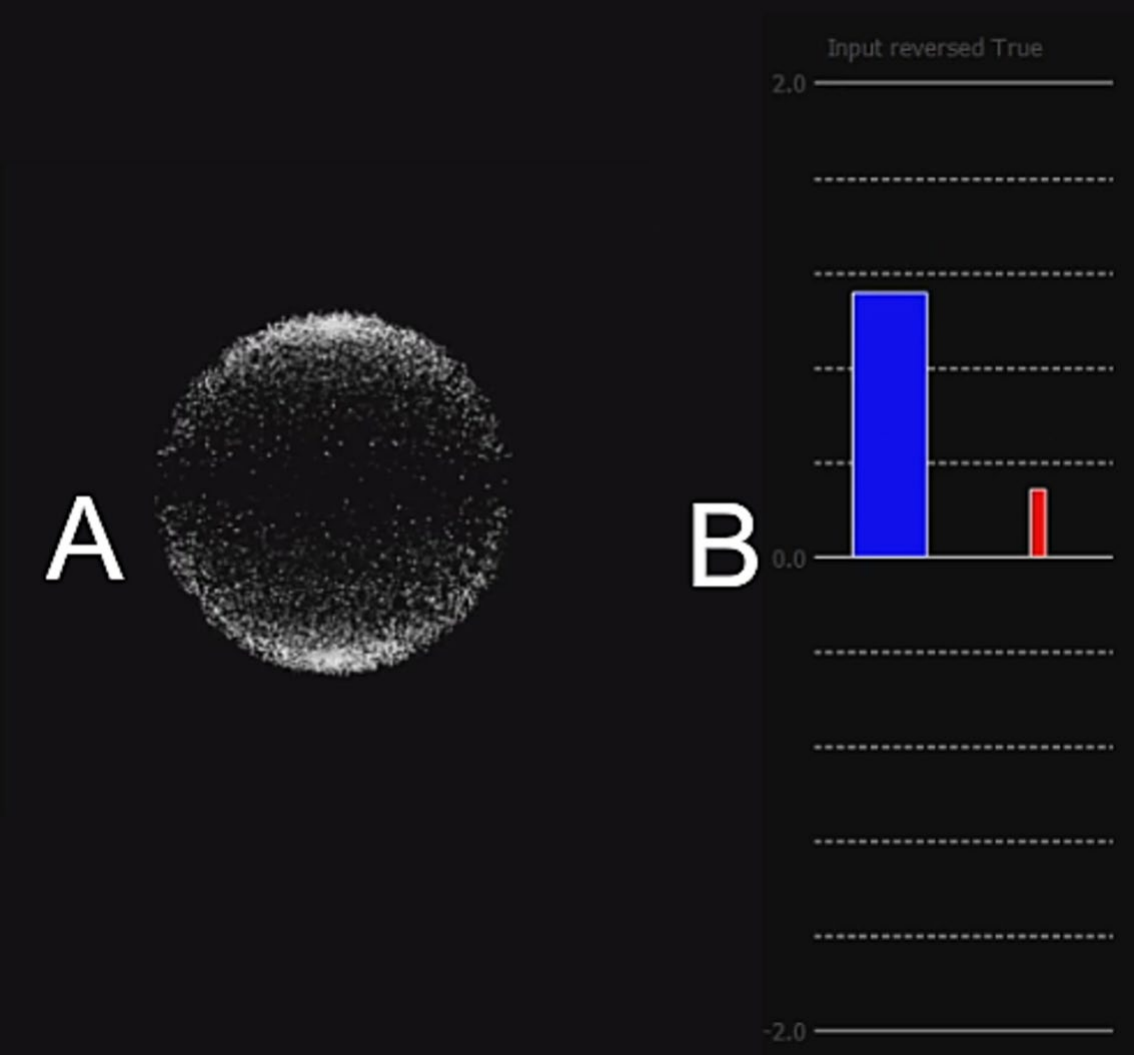
**(A)** Fixation image for the baseline run, animated. The white dots form a sphere, which slowly rotates clockwise around the vertical axis. **(B)** Bar feedback: the blue vertical bar originating at 0 indicated the current NF *Z*-score on a graduated axis ranging from 0 to 2 (positive NF) or –2 to 2 (full NF). The thinner red bar on the right-hand side translated artifact detection, multiplicated by 1.96 to scale with the blue bar. The Y-axis was reversed to transpose alpha power desynchronization into a blue bar going up. Participants could not see numbers nor deduce that the axes were swapped.

The *Z*-score was transformed into visual feedback as a blue bar of about 2 cm wide on a black background (see Figure 5). A horizontal axis indicated the threshold value at 0 and vertical axis had graduations from −2 to 2 without any numbers displayed. The vertical blue bar represented NF score, originating at the center of the graphical feedback. The coordinates of the vertical axis were flipped such that a negative change in power (i.e., TRPD) translated into a bar going up. About half a centimeter to the right a thinner red-colored bar displayed the artifact detection during the current epoch. Its value ranging between 0 and 1 was multiplied by −1.96 to convert it into the coordinate space, hence moving upward and proportionally to the feedback.

#### Artifact feedback

For artifact detection during trials, the current 2 s epoch was bandpass filtered between 1 to 40 Hz and then was split in 18 chunks of 200 ms with 10 ms steps. Each of these “sub-epochs” received a weight corresponding to their rank. The most recent sub-epoch had a weight of 18 while the oldest had a weight of 1. The sum of all sub-epochs crossing the threshold multiplied by their weight was divided by the sum of all possible weights (i.e., 171). This method returned an artifact score between 0 and 1 for the whole epoch. When presented as a one-dimensional bar, the feedback gave the impression of artifacts being emphasized at first and then diminishing over time. Artifact detection used a 100 μV threshold, which was more sensitive than the artifact rejection threshold applied on baseline recordings and during offline analysis (i.e., 150 μV).

### Offline data extraction

#### NF pipeline

The real time NF values for PSD and *Z*-score were saved directly in the EEG data files. Although there were artifact-reduction methods (i.e., ASR and CSD in Part 1 and CSD in Part 2), we wanted to prevent artifacts in the analysis and removed EEG artifacts segments via threshold method and interpolated bad channels. Hence, for offline analysis we applied a 150 μV threshold filter on baseline and NF runs, split into 2 s epochs. This artifact rejection occurred after bandpass filtering and before ASR and CSD algorithms. If more than 33% of the epochs were flagged as artifacts, there was an extra step during which all channels present in more than 33% of the epochs were interpolated via spherical spline interpolation (using “interpolate_bads” from MNE), and artifact rejection took place including reconstructed channels. The offline analysis only comprised artifact-free epochs in both baseline and NF runs.

To simulate the potential results of Part 2, i.e., using a similar setup with a reduced number of electrodes, we repeated the offline analysis with 9 channels only.

#### Inverse connectivity estimates

Inverse connectivity was computed for sessions 1 and 10. Electrode montage was provided by the EEG cap manufacturer for all channels. Only signals during feedback presentation of baseline and NF runs were used for computing inverse connectivity. Raw EEG signal was re-referenced to common average reference then bandpass filtered using Butterworth filters between 0.5 to 40 Hz of order 5 and 4, respectively. Then epochs of 2 s were extracted and referenced to the first value of each epoch.

The “fsaverage-5120” reference model from freesurfer (Fischl, 2012) was used to provide a generic model of the brain for source space computations. The source space was initialized with parameters “oct6” and “pial.” We used the dSPM method from mne-connectivity (Gramfort et al., 2013) and “PALS_B12” atlas reference annotations enabling to extract surface activity from Brodman areas 9 (DLPFC), 7 (SPL) and 23 (PCC) in the alpha range. The baseline epochs were used to train the noise covariance matrix. The connectivity analysis was performed on NF epochs using weighted phase lag index (wPLI, Vinck et al., 2011).

### Statistical analysis

#### Changes in parietal alpha activity

We collected 2 dependent variables on which we ran ANOVA or *t*-tests: PSD in dB units and *Z*-score in SD units. For assessing the change in NF ability over time (hypothesis a) we computed a 2×2 RM-ANOVA of PSD with ‘run type’ (baseline vs. trials) and time (sessions 1 to 10) as within factors. This model also allowed for investigating long-term changes in baseline parietal alpha actitivity (hypothesis d) via the interaction between factors run type and time. When analyzing *Z*-scores, which required baseline runs as a reference, we only compared the dependent variables by time, so we computed an RM-ANOVA of *Z*-score with time (sessions 1 and 10) as within factor and used the model to evaluate average *Z*-score regardless of session using a one sample *t*-test. To check whether the presence of feedback influenced *Z*-scores (hypothesis b), we ran a 2×2 RM-ANOVA of *Z*-score with presence of feedback (feedback and no feedback) and time (sessions 1 and 10) as within factors. For assessing the difference of expressivity in the feedback (hypothesis c) we ran a 2×2 RM-ANOVA of *Z*-score with feedback expressivity (positive only feedback and full feedback) and time (sessions 1 and 10) as within factors.

For computing the within-factors repeated measures type III ANOVA models, we used the R package afex1.3–0 via the function “aov_ez.” In case of missing values we used “aov_4.” Regardless of the model, the random effects structure was maximally defined unless stated otherwise. Post-hoc tests were performed using the package emmeans1.9.0 which allows for comparing adjusted group means.

For investigating learning effects according to the power law (Logan, 1988, 1992). We used a log–log transformation, i.e., a log transformation of the dependent variable and the independent variables, hence fitting linear regression models after the following formula:


(1) $$\mathrm{model}=\log\left(Z\right)\sim \log\left(\mathrm{session}\right)$$

For the log transformation to work in the case of *Z*-scores (logarithm transformation fails when <= 0) we shifted all *Z*-scores above zero by adding the minimum *Z*-score plus ε . Hence, before transformation we applied the following formula:


(2) $$\begin{array}{l} Z_{pos}=Z+|Z_{\text{min}}|+\varepsilon \;\\ \mathrm{with}\;Z_{\text{min}}=\min\left(Z\right)\;\mathrm{IF}\;\min\left(Z\right)<0\;\mathrm{ELSE}\;0\;\\ \mathrm{and}\;\varepsilon =.01 \end{array}$$

After fitting the statistical regression model, we extracted predicted values for each session. These values, computed in the log–log space were transformed back to the original scale using the inverse formula, resulting in a learning curve over time:


(3) $$Z_{\mathrm{predicted}}=\exp\left(\mathrm{Zpredicte}d_{\log-\log}\right)-|Z_{\text{min}}|-\varepsilon$$

The significance of individual log–log regression models was used to assess whether NF training followed a learning curve at the individual level. Also, we individually computed one sample *t*-tests to check whether *Z*-score was different to 0, respectively, for session 1 and 10.

#### Connectivity in the alpha and theta ranges

The inverse connectivity estimates were compared using paired *t*-tests. To detect differences due to NF training we compared sessions 1 and 10. For each of the 600 epochs per participant and session we compared connectivity estimates between BA 7, 9 and 23, respectively, lateralized for right and left hemisphere (e.g., DPLFC-Right vs. PCC-Left) resulting in 15 pairwise comparisons. These comparisons were computed for the alpha and theta frequency ranges, respectively. Significance levels were adjusted for multiple comparisons.

#### Behavioral data

Since this was the first part of the study, we could perform an exploratory correlation. While one might see it as inflating false discovery rate, any significant finding would require replication in the second Part reported here. We exploratorily compared *Z*-score with an array of tests: (1) the self-reported visual analog scales nausea, unease and presence, (2) the general presence (GP) item and subscales of IPQ called involvement (INV), spatial presence (SP) and experienced realism (REAL); (3) the subscales of FKS concern flow and process. Each subscale was tested against *Z*-score using Spearman’s correlation coefficient. These subscales were entered in Wilcoxon signed rank tests (for paired samples) between session 1 and session 10. For testing the correlation between fatigue and baseline alpha power (hypothesis h), we ran a Spearman’s correlation test between PSD during baseline runs and the rating in VAS fatigue before recording the baseline.

#### Strategies and self-reports

At the end of NF sessions and answering questionnaires, participants were instructed to write down how they attempted to modulate their brain activity and whether the method or strategy used changed across the sessions.

## Results Part 1

All participants completed the 2 VR + 10 NF sessions. One participant was excluded for using feet, thumbs and full body movements over 3 different sessions despite being asked repeatedly not to do so. Of the remaining *n* = 9 participants *n* = 5 were female. Participants had a mean age of 25.4 (SD = 4.2) years and five were enrolled in an undergraduate psychology curriculum. As planned, all participants completed the training within 3 weeks. During the first EEG recording of participant 1 in session 1, the cables of two sets of 16 channels were inverted resulting in 32 channels being swapped. Since it was the first session and did not include Pz, we decided to keep the participant and rectify the issue by reordering the channels during offline analysis. Note that channel positions were rectified in the shared dataset too.

### Neurophysiological results

**Figure 6:**
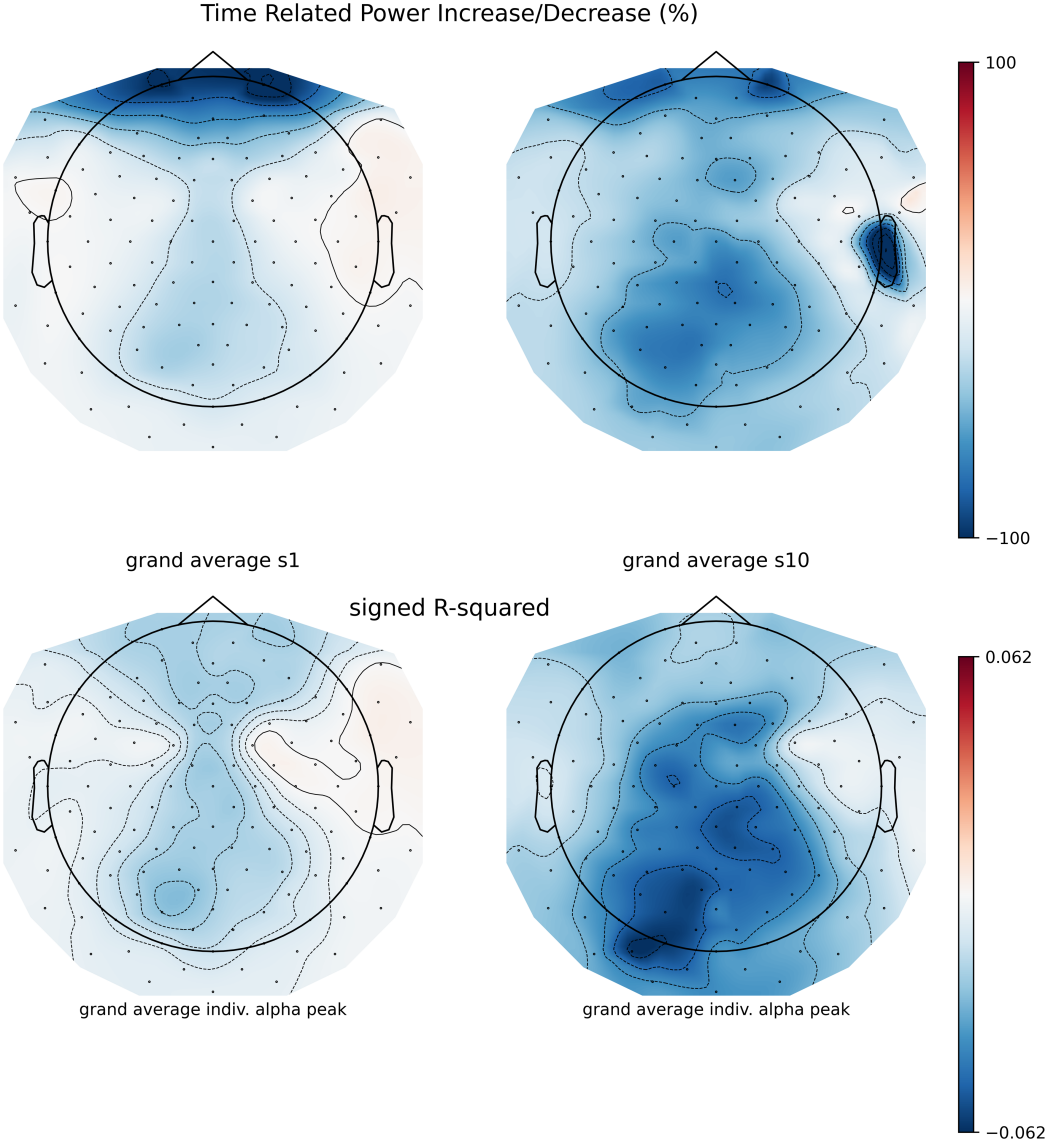
Session 1 and session 10 time-related power increase/decrease (TRPD/I) from artifact filtered runs to their reference baseline of *n* = 9 participants, no CSD filter was applied. Before TRPD/I computation (top row), power during 2 s epochs during NF was compared to baseline epochs using signed *r*-squared (bottom row).

**Figure 7:**
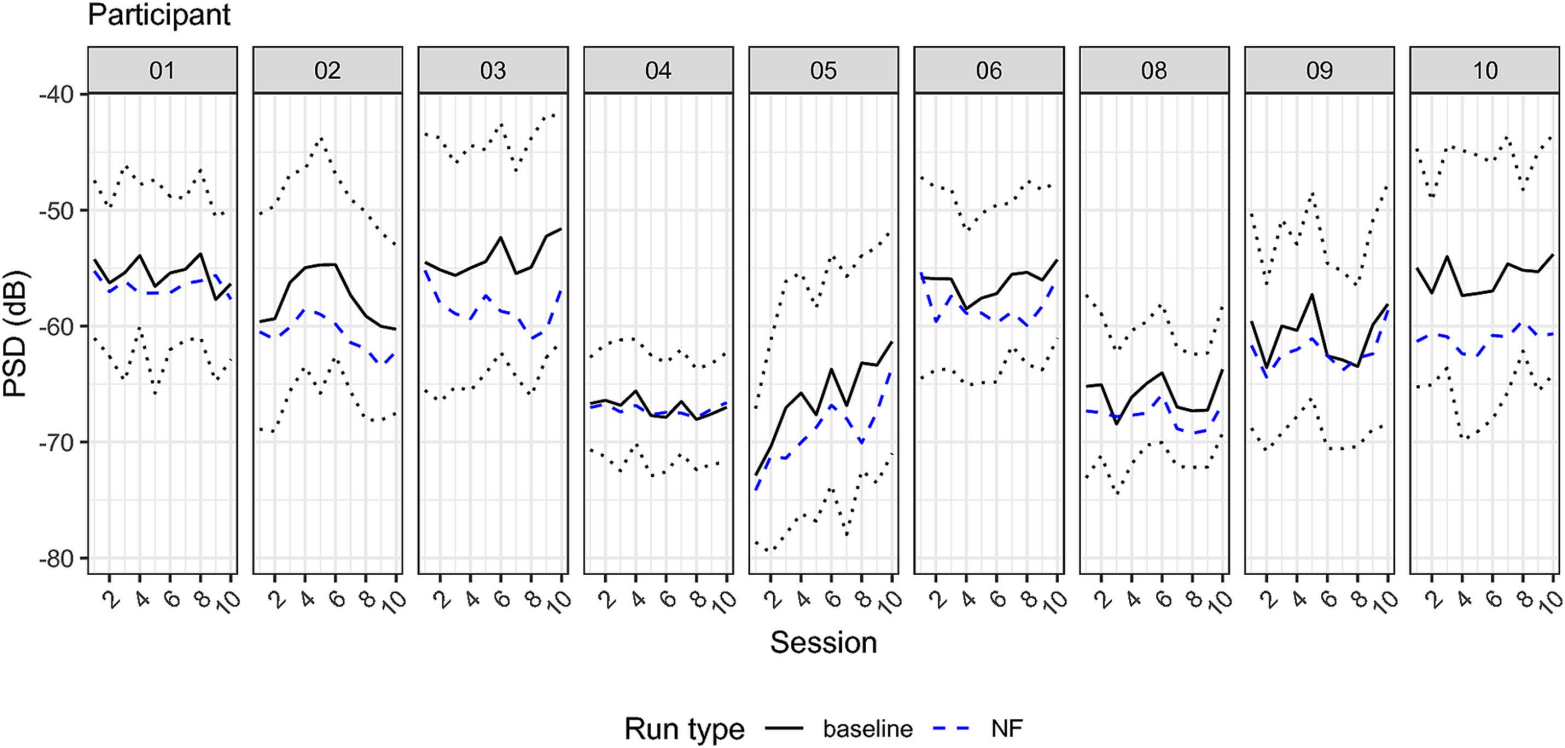
Individual power spectrum density across sessions at the individual alpha peak. Solid line indicates the baseline average PSD, the dashed line PSD average during NF trials. The dotted line indicates 1.96 times the standard deviation of baseline PSD comprising 95% of the distribution, and hence the range of the visual feedback. Note that the feedback was reversed, training participants to reduce PSD.

To assess whether NF training leads to changes in parietal alpha power, we entered the PSD (dB) values in a 2×2 RM-ANOVA using time (1 and 10) and run type (‘baseline’ and ‘NF’) as within-subject factors. The ANOVA revealed a main effect of run type (*F* = 9.92, df = 8, *p* = 0.014, generalized eta squared ges =0.035). Post-hoc estimated marginal means from the model showed an average PSD of −59.4 dB for the baseline and 61.5 dB for NF trials showing a significant reduction of 2.03 dB, *t* = −3.52, 95% CI [−0.54, 3.52]. Neither the main effect of session nor the interaction was significant. For visualizing PSD across sessions, see Figures 6, 7.

For the dependent variable *Z*-scores an RM-ANOVA (equivalent here to a dependent *t*-test) was computed with within-subject factor time (sessions 1 and 10), which revealed no significant effect of time. From the trained model, in a post-hoc analysis, we tested *Z*-scores against 0 (function “test” of package emmeans for a one sample *t*-test) and found an overall significant average *Z*-score of −0.485 (df = 8, *t* = −3.91, *p* = 0.0045) independent of session number. These results validate that participants were able to modulate their parietal alpha activity (hypothesis a), however, at the group level there was no significant improvement in performance over time.

#### Individual differences

**Figure 8:**
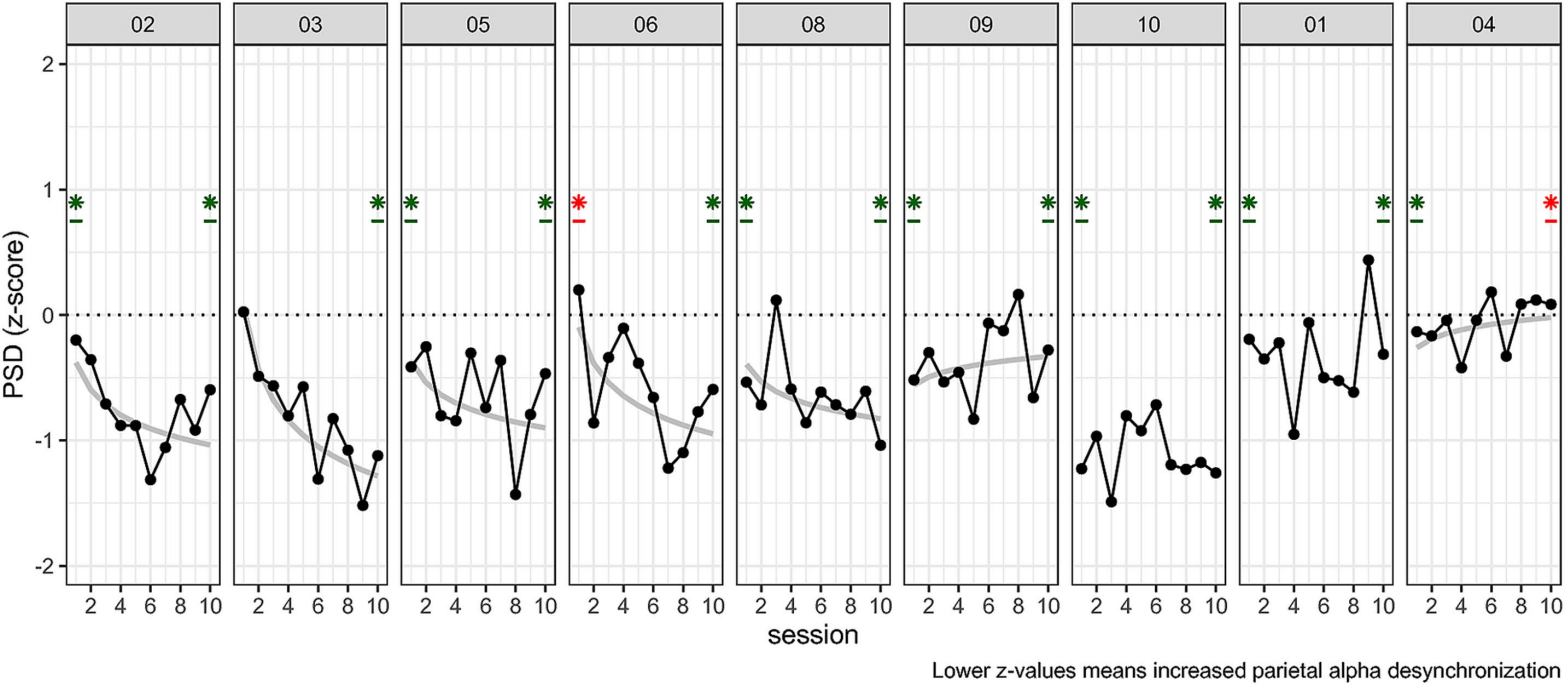
Individual performance across sessions 1 to 10 for *Z*-scores. Participants were reordered for better readability (5 Learners, 3 stable performers, 1 non-responder). Plots also *t*-test against 0 at session 10 for which significance is indicated with a star. The color indicates better than random average (green) or worse than chance level, i.e., regulation in the wrong direction (red). Plots also display the log–log learning curve only if significantly fitting (grey) at H_0_ < 0.05.

As we found a reduced alpha power in NF trials as compared to baseline, but no effect of time, we investigated individual time courses to see whether learning occurred for some participants. To test this, we ran individual one sample *t*-tests of *Z*-score at session 10. For investigating whether learning occurred we fitted individual power law models (see Equations 1, 2 and 3) from the *Z*-score. The one sample *t*-tests showed that *n* = 8 participants performed above chance level at session 10. For learning we were expecting negatively sloped curves to reflect a decrease in peak alpha power. We identified two stable performers, who had non-significant learning curves but were above chance level at session 10. There were 5 learners, showing negative curves with above chance level at session 10 (a negative curve indicated an improvement in NF modulation). The two remaining participants had a positive curve. But one was better than random at session 10 while the other was also significant but both in the wrong direction (i.e., participant 4, see Figure 8).

#### No feedback (transfer) trials

For assessing the difference between NF and transfer trials (every 10^th^ trial) we used an RM-ANOVA of *Z*-scores within time (sessions 1 and 10) and NF (with and without). The results indicated no significant difference when feedback was removed, nor any main effect of session or interaction between NF and time. It validates hypothesis b, meaning that regulation is possible without NF.

#### Positive-only-feedback trials

To assess the difference between positive-only-feedback and full-feedback, run 1 (positive) and 2 (full feedback) of sessions 1 and 10 were selected and entered a 2×2 RM-ANOVA with *Z*-scores as dependent variable and run and time as within-subject factors. The ANOVA yielded no significant main effect or interaction, validating hypothesis c.

#### Inverse connectivity

None of the paired *t*-tests for connectivity between lateralized BA areas 7, 9 and 23 yielded a main effect of time, before and after correction for multiple comparisons. Thus, we did not find changes in fronto-parietal coupling in the alpha and theta ranges.

### Behavioral results

For testing the correlation between parietal alpha PSD during baseline and VAS fatigue, we extracted all baseline PSD averages and VAS fatigue during all 10 sessions of 9 participants. We used a Spearman correlation test which returned a significant positive correlation between both outcome variables. (*ρ* = 0.209, df = 0.88, *p* = 0.048). Thus, hypothesis h was confirmed.

All participants successfully completed the pre and post VR session. The main source of breaks in the feeling of presence encountered by participants was due to the inability to pass doors and obstacles while navigating in VR, participants had to “return to their feet” which required interacting with the experimenter to trigger the event via the control panel. Other interruptions of the feeling of presence were due to questions when instructions were not fully understood or (rare) technical issues. These breaks in presence (BIP) were rated by the experimenter on a likert scale from 0 (no interruptions) to 3 (a lot of interruptions), The experimenter rated an average score of 1,33 per session, SD = 0.66. During the post VR session, the BIP score was reduced to 0.77, SD = 0.91.

Behavioral test results showed an overall high sense of presence in IPQ general presence, high realism (VAS realism) accompanied by low discomfort and nausea scores. Scores of all behavioral measures are provided in Table 1. Exploratory paired Wilcoxon signed rank tests comparing pre and post VR in the subscales of IPQ, FKS and the VAS scores, did not yield significant differences. The results of the Wilcoxon signed rank test of general presence (IPQ) revealed no significant improvement in the sense of presence between pre VR exploration session and post VR exploration session, which confirmed hypothesis f.

**Table 1:** Mean and SD of all questionnaires and subscales, between the first session of VR (PRE) and the last one that occurred after training (post).

		VR pre		VR post	
		Mean	sd	Mean	sd
IPQ	Spatial P. (Spatial presence)	1.96	0.57	1.76	0.69
	Involvement	1.58	0.64	1.56	0.91
	Realism	0.08	0.99	−0.08	1.02
	G. Pres. (Spatial presence)	2.11	0.78	2	0.71
FKS	Flow	55.22	6.96	55.22	6.72
	Process	11.56	2.92	10.89	2.32
	Concern	5.11	2.15	5.67	3.16
VAS	Realism	7.89	1.2	7.56	1.07
	Unease	1.78	2.25	1.22	1.81
	Nausea	1.44	2.06	1.67	2.05

Abbreviations: Spatial P. = Spatial Presence, G. Pres. = General Presence.

For assessing correlations between changes in behavioral scores and changes parietal alpha modulation, in an explorative way, we extracted pre-post differences of the behavioral variables and tested using Spearman’s correlation test with the difference in average individual *Z*-score in alpha power between session 1 and 10. No significant associations were found.

### Strategies

Most frequently, relaxation techniques based on breathing were used by participants 1, 2, 8 and 9 but unsuccessfully for number 4. Also, participants 1, 2, 4, 8 and 9 used more complex tasks such as mental calculus or planning during the first session but abandoned them immediately on session 2 except for participant 4.

Participants used various strategies, often starting with mental tasks that increase mental workload or requiring concentration such as counting, visualization and planning, then moved on to more internally oriented sensory tasks such as breathing or motor imagery. We described individual strategies in Supplementary Table S1 along with a description of the average NF performance obtained by their individual learning curve and one sample *t*-tests against 0 at session 10.

### Online versus offline results

To assess the effect of reducing channels requested for Part 2 of this study, the average PSD of every NF run was entered in a 3×2 RM-ANOVA with method (online, offline and offline 9 channels) and run type (baseline and NF) as within subject factors. The ANOVA yielded a significant interaction between method and run type (*F* = 9.38, df = 12.91, *p* = 0.004, generalized effect size ges < 0.001), a main effect of method (*F* = 252.44, df = 15.88, *p* < 0.001, ges = 0.054) and a main effect of trial type (*F* = 16.71, df = 8, *p* = 0.003, ges = 0.003). Post-hoc analysis was conducted without adjusting for multiple comparisons, indicating that the PSD from the online trials did not differ from offline trials, but was significantly lower online during baseline with a difference of −0.362 dB, 95% CI [−0.647, 0.077], df = 25.7, *p* = 0.015. Compared to the online pipeline, the 9-channel subset was significantly lower in both baseline and trials with an average difference of −2.29 db, CI [−2.54, −2.03], df = 16, *p* < 0.001. Also, compared to the offline pipeline, the 9-channel subset was also significantly lower with −2.37 dB, CI [−2.63, −2.12], df = 16, *p* < 0.001.

We performed the same comparison using *Z*-scores in a RM-ANOVA with method (online, offline or offline 9 channels) as within-subject factor. The ANOVA returned a main effect of method (*F* = 9.63, df = 14.47, *p* = 0.003, ges = 0.063). Non-adjusted *post hoc* analysis indicated that offline *Z*-score was on average lower than online with a difference of −0.171, CI [−0.259, −0.084], df = 16, *p* < 0.001. Also, the 9-channel offline subset had a higher *Z*-score than the offline full channel set with a difference of 0.135, CI [0.048, 0.222], df = 16, *p* = 0.005 (for visually comparing signal processing pipelines, see Supplementary Figure S1).

These results indicate that offline analysis slightly deviated from online data when improved artifact rejection was applied, resulting on average in better NF modulation.

### Channel reduction

For assessing whether channel reduction still enabled NF alpha power reduction (hypothesis e), we ran a 2×2 RM-ANOVA of PSD with run type (baseline and trial) and time (1 and 10) as within variables. We found a significant main effect of run type *F* = 5.60, df = 8, *p* = 0.045 but no effect of time nor interaction. Post-hoc analysis showed a difference between baseline and trials of −1.44 dB, CI [−2.84, −0.037], df = 8, *p* = 0.045. Another RM-ANOVA of *Z*-scores with session as within variable returned no significant main effect of session. The hypothesis e is confirmed such that participants would be able to reduce their parietal alpha power also when provided NF training with a lower number of channels, however we found no training effect (see Supplementary Figure S1).

## Discussion Part 1

### Neurofeedback

#### Ability to modulate

At the end of 10 sessions of NF training, all participants but one performed above chance level with respect to parietal alpha modulation. Regardless of session number, the PSD was lower during trials than during calibration. At the group level, participants were therefore successful at desynchronizing subject-specific alpha power in the parietal cortex but did not improve over time when comparing performance in sessions 1 and 10. After running an additional *t*-test at session 1 we found out that 8 participants were already above chance level before any substantial training occurred.

According to Logan’s instance theory of automatization learning follows typically a power trend with steep progress at the beginning and asymptotic performance with training (Logan, 1992). Therefore, we fitted the power trend to individual learning curves (*Z*-scores) and found it significant for *n* = 5 subjects, indicating learning, albeit not visible on the group level. We may speculate that with more training sessions more subjects would have learned to regulate their alpha activity. In the earlier days of applying neurofeedback for treatment of, e.g., epilepsy (Sterman, 2010) or for communication in locked-in patients (Kübler et al., 1999) NF training continued for several weeks up to months and years. Likewise, for treating ADHD in children and adults, the number of training sessions is considerably higher (e.g., 30 in Mayer et al., 2016).

#### Validation

To provide uninterrupted feedback to the participant during online NF, there was artifact detection but flagged signals were not rejected. We assessed whether there were differences in average alpha power between online and offline as large differences would impact perceived performance. Results indicated no difference in alpha power during the NF trials between online and offline trials but found a reduced power during online baseline trials. While online baseline trials already had artifact rejection, the offline analysis added interpolation of bad channels while being stricter with respect to amplitude threshold. This significant difference in alpha power subsequently translated into offline *Z*-scores being 0.17 lower than online scores, which translates to better NF performance offline than online. Still, results indicated significant difference in alpha power between baseline and trials in both online and offline pipelines. It directly demonstrates participants were able to modulate their activity via NF. Also, descriptively, when visualizing the *Z*-scores averages for every participant at every session we find very close offline and online results regardless of the artifact rejection method (see Supplementary Figure S1).

#### Strategies

We noticed a recurring pattern in the strategies used for modulating parietal alpha. Five out of the nine participants used a strategy related to breathing, either breathing deeply or even withholding their breath. Although breathing worked well for some subjects, it is not what we were aiming at with NF as it is a strategy using voluntary bodily changes instead of mental imagery. Overall, we could not identify any specific mental strategy that was particularly effective or commonly used by participants.

#### Channel reduction

As mentioned in the validation section, there was a significantly better average *Z*-score in the offline artifact filtered pipeline as compared to the online average, but this was found in baseline trials and not the case during online NF trials, showing that participants were successful at keeping their artifacts low, and that the system could operate well in real-time. The offline analysis with reduced number of channels yielded reduced performance (*Z*-scores), but alpha power was still reduced. Individual offline learning curves remained similar and only one participant remained on chancel level. We may speculate from these results that extended NF training might be necessary for successful parietal alpha power down regulation. Part 2 of this Study investigated performance when providing participants with NF from 9 channels only.

### Behavioral results and VR experience

Although subjects successfully reduced their parietal alpha power, we found no effect on the sense of presence. This may be because we did not instruct them to re-use their strategies applied during NF sessions. The number of sessions was presumably too low to evoke any longer lasting effects on parietal alpha. Furthermore, the sense of presence was already rated high at the beginning such that it might have been difficult to further increase it with NF.

### Connectivity

We hypothesized decreased frontal inhibition on parietal cortex when experiencing presence. The connectivity analysis, however, did not return any significant difference in connectivity between the dorsolateral prefrontal cortex (BA-7 SPL) and the parietal cortex (BA-23) between session 1 and session 10. This is in line with the behavioral results.

### Limitations

Neither a control group was included nor an inverse task was applied that would have allowed us to draw conclusions about the specificity of found effects. However, as we were aiming at exploring learning of parietal alpha modulation and its potential effect on the sense of presence, a control group was not compulsory at this point. With *N* = 10 the sample size was small and with 10 sessions training time short. Traditional NF studies comprised many more sessions (Arns et al., 2014) providing more time for learning. However, the late start of the project (due to the Covid-19 pandemic) with no extension of funding, did not allow for longer training time. Further, we did not encourage the participants to apply their strategies during moving in the VR environment. Having said this, in the realm of BCI research 10 sessions are a considerable number of measurements allowing for within subject analysis of learning effects.

## Conclusion Part 1

To conclude, participants were able to modulate their parietal alpha activity when being provided with NF in comparison to the baseline activity. Such modulation was also detectable offline with a reduced number of channels. They felt immersed in the VR world presented albeit independent of NF training.

## Part 2

### Introduction

The development of NF is historically closely related to progress in brain-computer interfaces (BCIs). With EEG, magnetoencephalography (MEG), functional magnetic resonance imaging (fMRI), or near infrared spectroscopy (fNIRS) signals as input (Soekadar et al., 2021), BCIs can enable control over an application such as a robot, neuroprosthesis, or communication and interaction software (Kübler et al., 2014) via different approaches to detect changes in brain activity. There are different levels of active participation of the user for generating a control signal (Zander and Kothe, 2011). One of them is the active modulation of the targeted brain activity. NF fosters learning to modulate specific components of the EEG to improve or restore brain functions via neuroplasticity (Loriette et al., 2021). Prior to the widespread use of HMD devices, most EEG-VR studies were conducted on stereoscopic 3D monitors, walls or at a larger scale on cave augmented virtual environments (CAVE) but without head tracking. Reviews including BCI-VR (Lotte et al., 2012) and NF-VR (Wen et al., 2021) studies highlight that VR provides a more fun, engaging and motivating experience and requires shorter training than conventional paradigms presented on a simple 2-dimensional video screen, while enabling recovery of brain functions in clinical groups.

EEG-VR studies have raised new challenges and limitations (Kober et al., 2017a), notably the increase in signal artifacts due to movements and extra equipment placed on the head (Choi et al., 2023) or the issue of cybersickness for which a NF study (Berger et al., 2022) attempted to influence its identified correlates (Chang et al., 2020; Krokos and Varshney, 2022).

In Part 1 we have demonstrated that it was possible to modulate parietal alpha power in real time via NF. In Part 2 we aimed at evaluating (1) the feasibility of translating the same NF protocol into VR, and (2) how the reduced set of 9 electrodes would affect downregulation of parietal alpha power. We formulated the following hypotheses:

- NF modulation is significant after 5 sessions
- Baseline alpha power is different between projector and fountain feedback presentations
- gel-based electrodes provide better signal stability leading to less artifact rejected trials than sponge-based electrodes
- Participants can modulate their parietal alpha power during transfer sessions
- NF learning follows a power trend according to Logan’s instance theory of learning (Logan, 1988)
- NF modulation is associated with increased sense of presence during training and transfer sessions.

### Materials and methods

#### Participants

We aimed at including *n* = 15 participants. To determine whether the sample size was large enough to replicate Part 1, we used calculations of the main effect of trial type (baseline versus trials) in PSD using gPower, with the following requirements: 5 training sessions, partial eta square of 0.035 (note that we used the more conservative generalized eta square from the results of Part 1), correlation among repetitions of 0.4 and only 1 experimental group. From the 10 participants suggested we increased the sample to 15 for a total number of training sessions of 75. Data collection was around mid-2021, and thus, still under the influence of the pandemic. For this reason the number of sessions was reduced from 10 to 5. One participant dropped out due to a COVID-19 infection. Hence, all results are based on a sample size of *n* = 14 participants (mean age 24 years, SD = 1.93, 10 female) performing 5 sessions of NF training. None were participants in Part 1. In this study, we prioritized the number of participants over the number of training sessions. All participants were trained for NF. Thus, no control group was investigated in Part 2.

#### Setup

**Figure 9:**
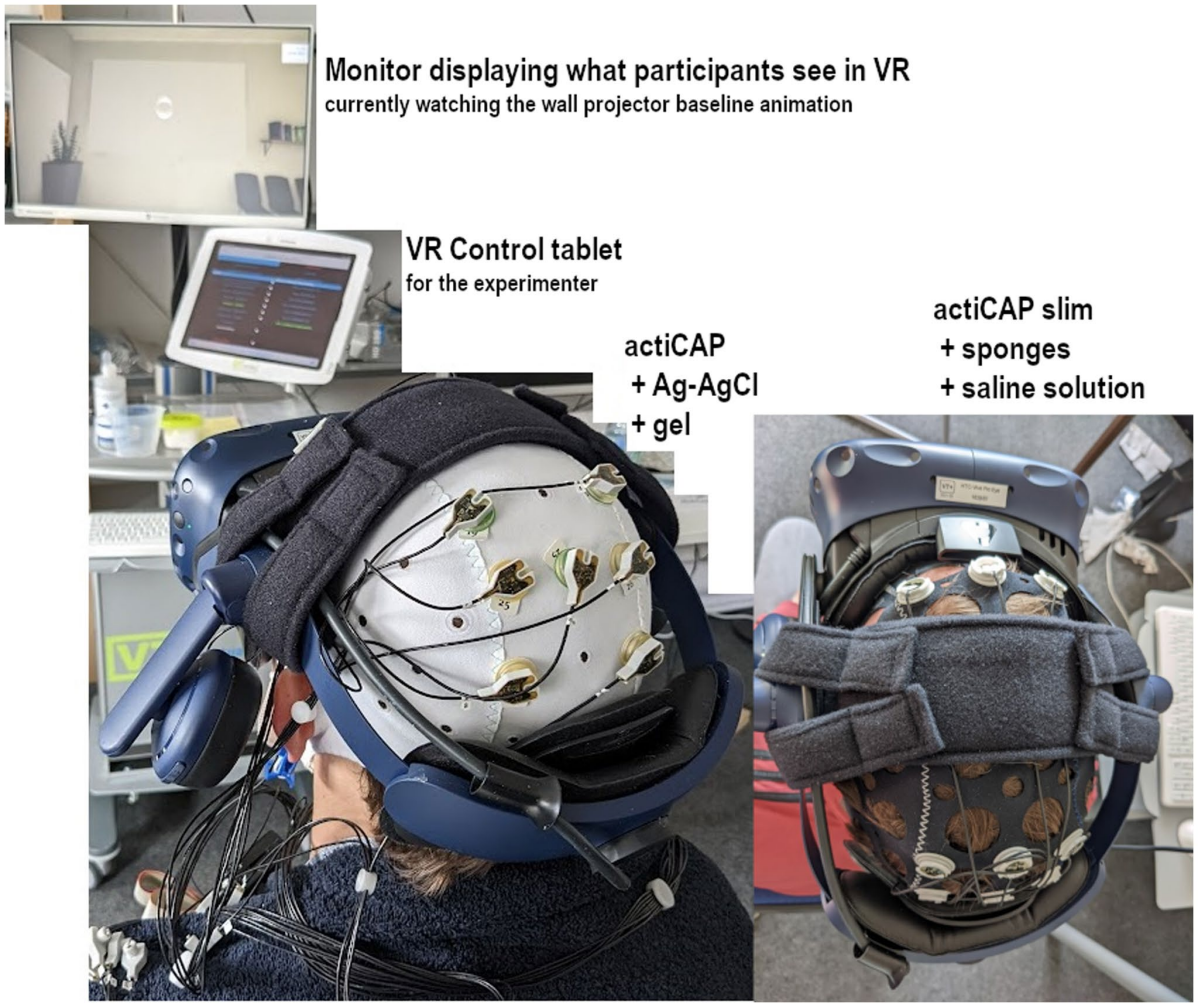
Participant seated and equipped with 9 channel EEG caps (left: actiCAP with silver-silver chloride electrodes and electrolyte gel; right: actiCAP slim with sponges soaked with potassium chloride solution (Brain Products GmbH) and HMD, currently attending the baseline measurement of the projector scenario). The monitor on the top shows what the participant currently sees, while the tablet monitor below allows the experimenter to control the current VR scenario. The longitudinal strap of the HMD is replaced by a comfortable lateral strap that provides room for midline Fz, CPz, Pz, and POz channel locations.

A sponge-based system with active electrodes was used that does not require extended preparation times and washing the hair after participation. While prioritizing convenience, we used a traditional gel-based system once to compare the two. For the gel-based system 9 active electrodes (actiCAP, Brain Products GmbH) were used on their original cap and connected to a BrainAmp amplifier (Brain Products GmbH). Nine active electrodes were placed over cylinder-shaped sponges soaked in an electrolyte solution (potassium chloride and water) for enabling good skin contact. The electrodes were socketed on a different EEG cap that was more elastic (see Figure 9). Both gel and sponge-based systems used the same portable LiveAmp amplifier (Brain Products GmbH). Both gel-based and sponge-based montages followed the international 10–20 system (Nuwer et al., 1999). The reference (REF) and ground (GND) were Ag/AgCl electrodes placed on the earlobes, each at one side of an ear clip placed on the left earlobe and prepared for recording with electrolyte gel. The online transmission of EEG signals to the NF computer was granted by wireless Bluetooth connectivity.

To clear up space for channels on the parietal area, we replaced the longitudinal strap originally on the HMD with a lateral strap (KIWI design, Shenzhen, China). The lateral strap secured the HMD on the head with comfort and allowed for accessing all midline channels from Fz to POz. The HMD was then carefully placed on top of the EEG cap, and signals were checked again. The lock-in place mechanisms of the HMD that pressed the frontal pole area and the occipital areas together with cushion pads intended at stabilizing the HMD on the head also helped prevent the EEG cap holding sponges from sliding.

#### Virtual reality

The participant was seated on a chair in VR. After the HMD was carefully placed on top of the EEG cap on the participant’s head it remained fixed until the end of the training. In this study, the participant remained seated on a chair for the whole duration of the experiment. The high immersive VR world depicted two different scenarios.

**Figure 10:**
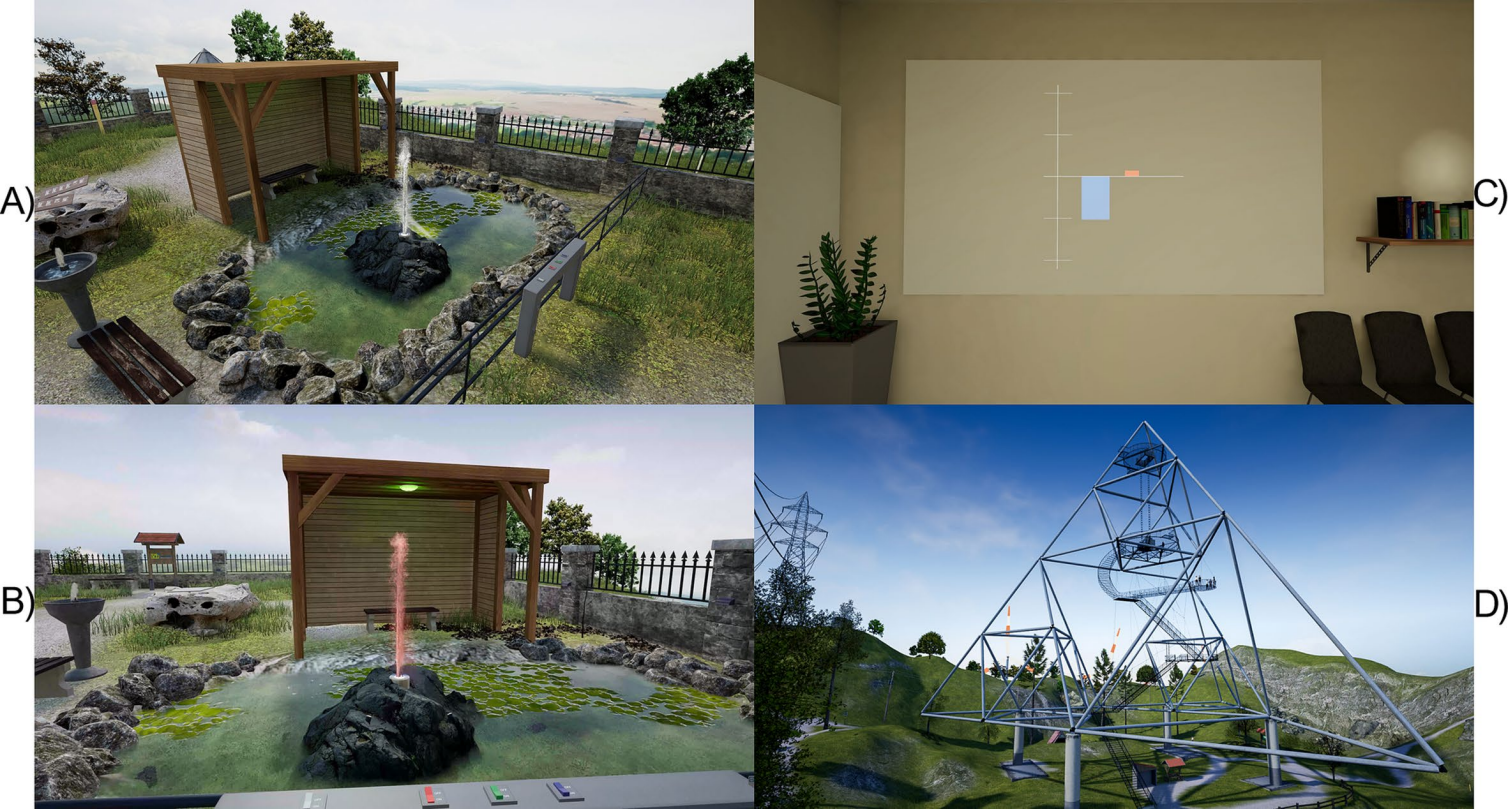
**(A)** Aerial view of the Fountain scenario in VR and **(B)** by the point of view of the participant. The water spray reflects a current *Z*-score of about −0.5. The wooden shed provides background contrast to the fountain. When the light at the top of the shed is green, the participants are required to modulate their parietal alpha activity. If artifacts are detected the water takes a red taint. **(C)** Projector scenario in VR. The NF blue bar is visible, currently reflecting a *Z*-score of about 1 (i.e., negative blue bar) and few detected artifacts (red bar). **(D)** “Tetrahedron” scenario for the transfer session. The participant could explore the VE using the paths but was not allowed to climb on the metallic structure (the scenario was originally built for exposure of patients with anxiety disorder).

The first scenario for NF training was in the same virtual location used in Part 1 and introduced two virtual scenes: an NF-controlled virtual projector indoors (Figure 10C), and NF-controlled fountain outside in the yard (Figures 10A,B). The experimenter pressed a button to move the participant (i.e., “teleport”) between the scenes. The first scene started at the back of an office room. In the “projector” condition the participant faced a wall screen that displayed a 2D bar feedback as similar as possible to the feedback that was provided on a monitor during Part 1. A vertical bar with an origin of 0 would move up or down depending on the NF score (Figure 10C). The second scene was a transcription of the bar feedback projected on a water fountain outside of the same building in the yard. In the “fountain” condition, the participant faced a fountain 3–4 meters away. The natural vertical spray of water could range to a height of about 3 m and would move instantaneously following the calculated NF values computed and transmitted at a rate of 10 times per second. When the NF value was minimal (*Z*-score of 1.96) the spray was almost invisible, in its rocky bed (see Figures 10A,B), at 0 it would be midway, and at −1.96 it would reach maximum height. By default, the spray remained mid-height at 0 to inform the participant of the “threshold” for which no further specific cue was provided in VR space.

After completion of the NF training, participants were invited once again to test the transfer of the skill in a second VR scenario. The scenario featured a VR walk on top of a hill in the countryside in the summertime with a panoramic outlook on farmlands (Figure 10D): Birds are chirping, and the sun is shining high in the sky with little to no clouds. A few middle-aged non-playing characters randomly walked the small paths and occasionally sat on the benches around the participants. There were 4 runs of either “exploration,” during which they were instructed to freely explore the environment or “transfer” during which they were instructed while exploring to continuously “move the bar up” like during the NF training but during an extended duration of 5 min. There was neither an NF-specific trial structure nor any feedback.

#### Behavioral measures

For evaluating sense of presence, we used again the IPQ scale at the end of every session. VAS for discomfort, nausea and sense of presence were assessed within the VE. Participants were asked via a pre-recorded voice to rate, using their voice, their level between 0 (not at all) to 10 (maximally). The audio was manually played from the experimenter who wrote down the answers.

#### Study timeline

The study comprised 6 EEG sessions each conducted on separate days within two weeks. The last session was a transfer session.

**Figure 11:**
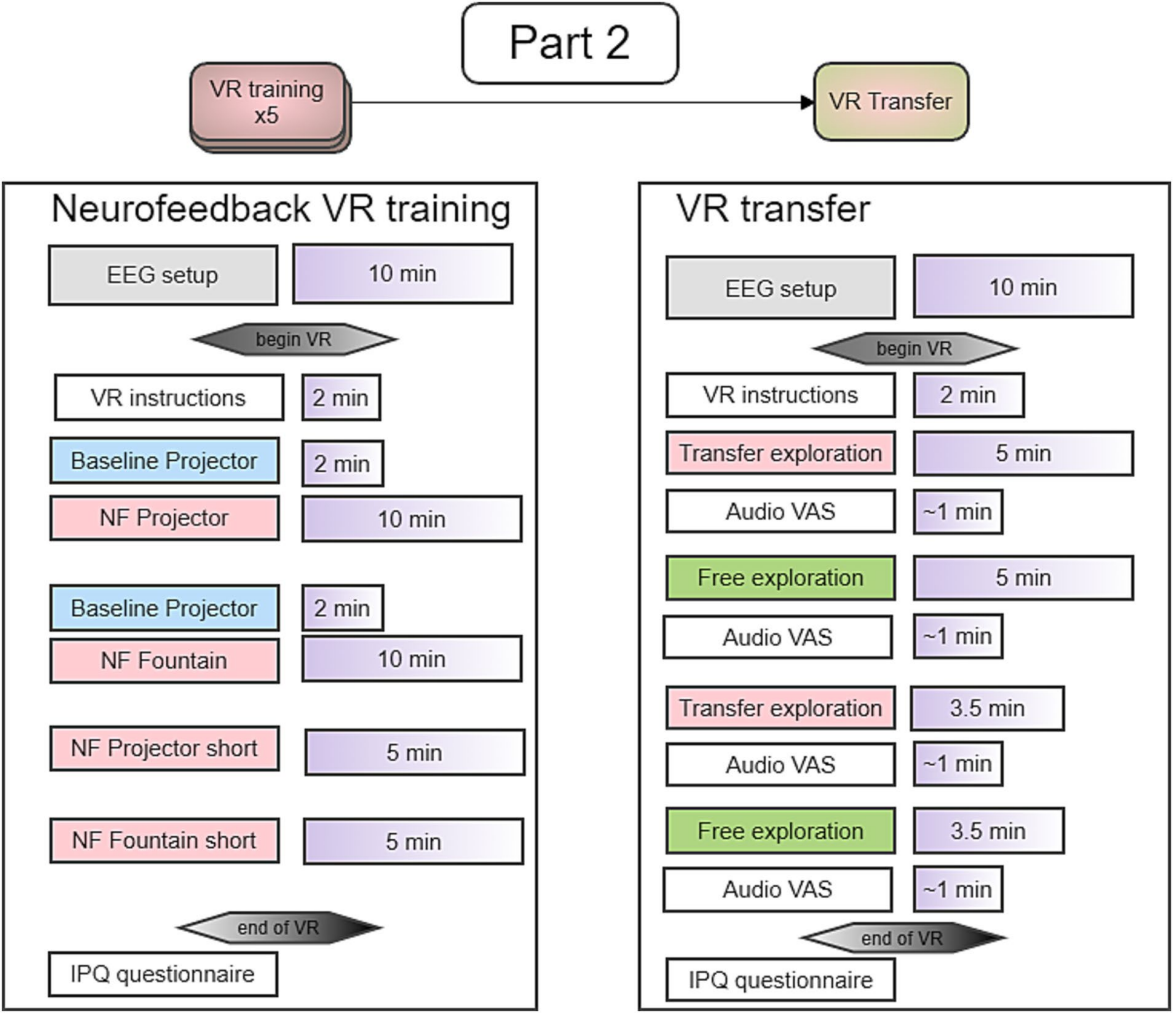
Timeline of Part 2 including 5 NF training sessions and a transfer session.

There were 5 sessions of NF in total. For sessions 1 to 4, we used a portable EEG system with sponge-based electrodes that was easy to set up and remove. To assess the signal quality of the sponge-based electrodes, we used active gel-based electrodes in the 5th session. The procedure for NF and transfer sessions are depicted in Figure 11. By alternating between feedback modalities, we could investigate short term effects of time within the session although the projector modality always came first.

#### Data extraction

The data extraction pipeline was adapted from Part 1. We reduced the number of input channels to 9 and tried to depart as little as possible from the methods used in Part 1. This pertains to the appearance of the feedback, the timings and the instructions provided to the participants, including not telling them about the real purpose of the study and not giving any indication about the direction of the power modulation. However, we added an artifact rejection when extracting mean and SD from baseline recordings. This artifact rejection specifically helped after noticing strong artifacts caused by movement. Also, to adapt to the low number of available channels, Artifact Subspace Rejection was disabled and the frontal channels only served for threshold-based artifact rejection. Hence, the current source density algorithm only used the signals from the 7 parietal channels. For baseline *Z*-score calibration and all the offline analysis, the artifact rejection threshold was lowered from 150 to 100 μV. As in Part 1, no artifact rejection was applied during real time feedback, only detection. Also, during offline analysis, after bandpass filtering we interpolated non-Pz channels if at least 33% of the epochs were flagged as artifacts on these channels, followed by artifact removal via the threshold method. Frontal channels AF3 and AF4 were dropped and CSD was applied on channels CP1, CP2, P1, Pz, P2, PO3, PO4. PSD from channel Pz was extracted using Welch’s method with nfft = 512 from which frequencies between 9 to 12 Hz were averaged. The decision to keep the CSD spatial filter even with a small number of channels seemed appropriate after validation from the simulated subset in Part 1.

For the data recorded during the transfer session, every run was split in 2 s epochs after bandpass filtering between 1 to 40 Hz. Epochs containing artifacts superior to 100 μV were rejected. We extracted the PSD of channel Pz for each epoch via Welch’s method, averaging power values returned between 9 Hz to 12 Hz.

#### Statistical analysis

Dependent variables were parietal alpha *Z*-scores and PSD. Independent variables were time (sessions 1 to 5), feedback modality (projector or fountain), run type (baseline and NF).

To simplify the statistical model, we compared PSD and *Z*-scores based only on the first and longest NF run of each feedback modality. For evaluating the success of NF training (hypotheses a and b), we ran a 2×2 RM-ANOVA with *Z*-values as dependent variable and session (1 and 5) and feedback modality (projector and fountain) as within subject factors.

To assess the absolute difference in PSD over time and between feedback modalities (hypothesis b), we computed a 2×2 RM-ANOVA of PSD during baseline with session (1 and 5) and feedback modality (projector and fountain) as within subject factors. Here we only considered PSD acquired during baseline to again simplify the statistical model.

Although the 2^nd^ runs were not considered for other statistics due to the increased complexity of including them in statistical models, we still used them to check for an eventual effect of time during sessions using a 2×2 RM-ANOVA of *Z*-score with runs (1 and 2) and modality (projector and fountain) as within subject factors for assessing hypothesis c.

For the transfer session in which participants explored the tetrahedron scene, they were asked to modulate their activity for several minutes. We checked changes in parietal alpha power between exploration runs with modulation and without modulation. For this post training session, the dependent variable was the parietal alpha PSD. Independent variables were task (transfer and explore) and time (run 1 and 2). We ran a 2×2 RM-ANOVA of PSD with time and task as factors to assess the difference between NF transfer and exploration (hypothesis f).

### Results

#### Neurofeedback

**Figure 12:**
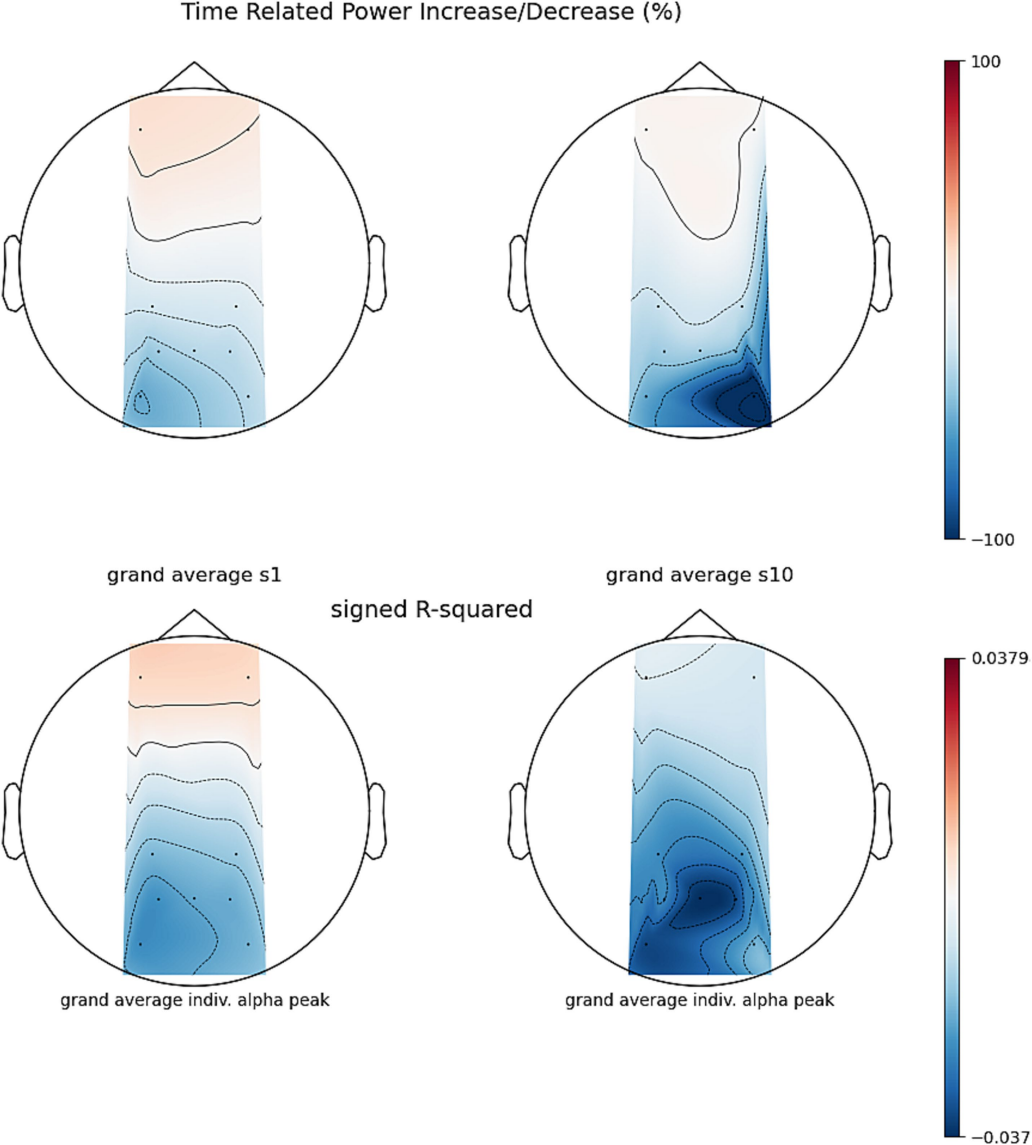
Session 1 and session 5, time related power increase/decrease (TRPD/I) between the PSD of artifact filtered runs and their reference baseline for *n* = 14 participants, no CSD filter was applied. Before TRPD/I computation (top row), power during 2 s epochs during NF were compared to baseline epochs using signed r-squared (bottom row).

To assess whether regulation of parietal alpha power increased over time, we modeled the NF *Z*-score in a 2×2 RM-ANOVA with feedback scenario (projector and fountain) and session (1 and 5) as within subject factors. We found a significant interaction between session and scenario (F = -4.79, df = 13, *p* = 0.047, ges = 0.097) and a main effect of time (F = -7.82, df = 13, *p* = 0.029, ges = 0.066). Post-hoc tests of the interaction revealed that the *Z*-score in the projector scenario decreased between sessions 1 and 5 by 0.51, (95% CI [0.19, 0.83], df = 22.9 *t* = −3.34, Bonferroni adjusted p_adj_ = 0.003). No significant change was found in the fountain scenario. Post-hoc comparisons of the main effect of session (averaged for both feedback modalities) showed a decrease of 0.23, (CI [0.05, 0.41], df = 22.9 *t* = −2.8, p_adj_ = 0.015). Note, that a significant decrease in *Z*-score translates to a better NF performance. This partially confirms hypothesis a that NF modulation improved significantly over time but was only found when training with the projector scenario. For visualizing changes in PSD over time, see Figure 12.

#### Baseline differences

The alpha PSD at electrode Pz during baseline was assessed via a 2×2 RM-ANOVA with feedback scene (projector and fountain) and training sessions (1 and 5) as within subject factors. It yielded no significant effects. This finding leads to rejection of hypothesis b, that feedback scenarios lead to different baseline values of alpha PSD.

#### Session-wise differences in the projector scenario

Pre-post results showed that there were only differences in the projector scenario. We further investigated the results by assessing pairwise differences between all sessions instead of sessions 1 and 5 only. After encoding the session factor with dummy contrast with the first session as reference, we calculated an RM-ANOVA on *Z*-scores which indicated only a marginally significant main effect of session (*F* = 2.47, df = 41.05, *p* = 0.073). Bonferroni-Sidak corrected pairwise comparisons of 4 dummy contrasts indicated *Z*-scores differing between session 1 and 4 (−0.53, CI [−1.03, −0.03], *t* = −2.71, df = 52, adjusted *p* = 0.036), and 5 (−0.51, CI [−1.01, −0.03], *t* = −2.62, df = 52, adjusted *p* = 0.045).

#### Learning curves

To assess learning, a power trend was tested on the 5 sessions (see formulas 1 and 4 in Part 1). For each feedback condition (projector or fountain) a linear mixed model (LMM) was trained to fit log(*Z*-score) by using log transformed session as numerical factor. It showed a significant main effect of log(session) (*F* = 11,45 df = 13, *p* = 0.005) in the projector scenario but not in the fountain scenario, meaning that the power trend significantly predicted session-wise performance across all participants, the factor was not significant in the model trained with data from the fountain scenario (see Supplementary Figure S2 for grand average plots). Learning curves support hypothesis e in the projector feedback condition.

**Figure 13:**
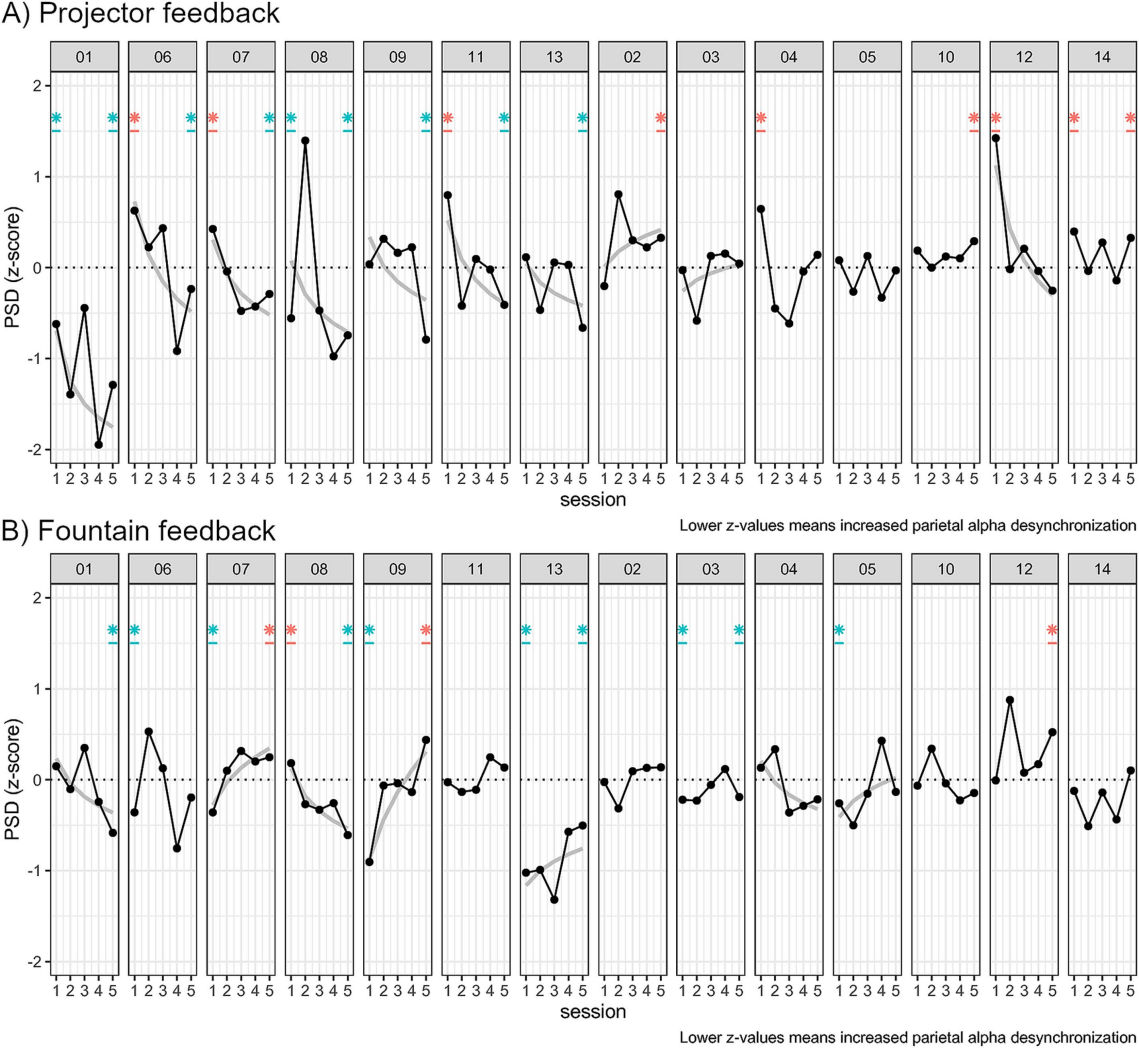
Plots of the average *Z*-score per session in the **(A)** “Projector” NF modality and **(B)** “Fountain” modality. Participants were grouped by their identification during “Projector” feedback: 7 learners with negative PSD log–log curves and significant *Z*-score session 5; 7 NF-inefficient without significant average *Z*-score. Log–log power law of learning curves are displayed only when significantly fitting at the *p* < 0.05 level. Asterisks at session 1 and session 5 are displayed when one sample *t*-test *Z*-scores different from zero at the *p* < 0.05 level during these sessions.

Individual regressions with log transformed *Z*-scores and session (see Formula 1) showed that 8 participants out of 14 had a significantly positive learning curve in the projector group (see Figure 13) from which 7 of them had significant *Z*-scores at session 5.

#### Intra-session performance

For investigating the stability of performance during a session, we ran an RM-ANOVA for *Z*-score by feedback scene, session and run. We found a significant main effect of run (*F* = 8.92, df = 13, *p* = 0.011, ges = 0.008) for an increase in *Z*-score between runs 1 and 2 of 0.091, (*t* = 2.99, CI [0.025, 0.158], df = 13, *p* = 0.011) revealing a decrease in performance between the initial run 1 following calibration and the shorter run 2. No further effects nor interactions were significant. Supplementary Figure S3 shows the difference between run 1 and run 2 across sessions.

### Sponge-based versus gel-based electrodes

To assess whether there was a difference in signal quality between sponge-based and gel-based electrodes, we ran a 2×2 RM-ANOVA of percentage artifacts rejected with run type (baseline or NF) and time (session 4 with sponge and 5 with gel) as within-subjects factors. We found no significant main effect or interaction (see Supplementary Figure S4), and reject hypothesis c.

### Transfer session

During the transfer session, participants reported re-using the “best strategies” previously used during NF training. Participants 7, 10 and 13 reported that it was challenging to simultaneously explore the VR environment and apply the NF strategy.

To assess whether participants desynchronized their parietal alpha during the transfer session, we entered PSD in a RM-ANOVA with time (run 1 and 2) and task (explore freely, explore with modulation) as factors. There was no significant effect nor interaction indicating a difference of PSD over time nor between transfer condition. Results did not support hypothesis d.

### Behavioral analysis

#### Correlation performance and sense of presence

To compare the sense of presence during the transfer session we ran a 2×2 RM-ANOVA of presence (“Anwesenheit”) with time (first and second run) and task (transfer and explore) as within-subjects factors. We found no main effect nor interaction. Therefore no change was found in the sense presence during the transfer sessions, hence rejecting hypothesis f.

We used the IPQ value collected at the end of NF training sessions to compare whether there was a correlation between *Z*-scores and sense of presence. Assessed separately for projector and fountain scenarios, we extracted the difference in performance between session 1 and 5, and the difference in self-reported IPQ scores between session 1 and 5. Results of correlation tests based on Kendall’s method indicated no significant association between change in presence and change in alpha modulation, rejecting again hypothesis f.

### Strategies

Strategies were written down at the end of each NF session after completion of the IPQ questionnaire. All participants reported the inner will to move the bar or fountain upward as strategy during NF. Other strategies were, e.g., mental calculus, visualizing and recalling memories or images, inner singing, imagining movements. One recurring strategy used by six participants was to think about one thing and ignoring distractions. Six participants used relaxation or focusing on breathing but only two kept it until the last NF session. Four of the learners employed inner voice in their strategy, by reciting the alphabet backward, either singing or thinking “up.” Participants 4, 6 and 8 reported using positive thoughts when upward movement of the bar or fountain was required, and frustration, anger or stress when downward movement was required.

### Discussion

Results of Part 2 showed that at the group level, participants were only able to increase their parietal alpha power in the projector condition. Individual analysis revealed that 7 out of 14 participants were able to increase the parietal alpha activity in the required direction. These 7 participants concurrently showed a positive learning curve despite the reduced number of sessions and channels.

None of the participants were so-called “fixed-performers” who modulated their parietal alpha power from the beginning, but did not improve over time. One may argue that the log–log regression curves (as seen in Figure 13) may not well fit to only five time points, but descriptively, they better fit the data as compared to the linear regression.

The *Z*-score increase from NF run 1 to run 2, suggested a negative effect of time on NF control, which might have been due to fatigue; no baseline was recorded prior to run 2.

The notable difference between projector and fountain conditions may be due to the easy interpretation of the classical bar feedback and that there was no visual distraction around it. Also, the zero level (i.e., no change to baseline) was easy to perceive as compared to the fountain scenario in which participants had to keep in mind that the zero line was at mid-height of the fountain. This may have increased the workload which in turn may lower performance (Käthner et al., 2014; Riccio et al., 2011; Zickler et al., 2013).

Similarly to Part 1, transfer trials were not significantly different from NF trials, indicating that in good performers the transition to real world conditions, in which no feedback is available, can be readily achieved. Real world application was also supported by the finding that the sponge-based electrodes, which are more comfortable, enabled training and did not lead to more artifacts as compared to gel-based electrodes.

As in Part 1, no specific strategy proved better than another. Overall participants used more active intentional strategies. Four out of 7 good performers used inner voice or language related strategies. One third of the participants used relaxation or breathing strategies which, however, were not maintained to the very end, very likely due to perceived ineffectiveness. This is somewhat in contrast to reports from Part 1, where participants tested active strategies and then switched to relaxation and breathing techniques after a few sessions. We may speculate that reduced external stimulation from the sound insulated cabin may have increased self-awareness in contrast to the stimulus rich and dynamic VR environment (e.g., fountain produced different noise depending on spray height), and that this may have fostered performance (Kober et al., 2017b; Xu et al., 2021).

The transfer session was globally successful at keeping participants engaged in the VE and all reported attempting to transfer their NF ability to move up a hypothetical bar or fountain. Yet the transfer runs during the transfer session did not lead to decreased parietal alpha activity as compared to non-transfer exploration runs nor any increase in sense of presence.

The targeted behavioral variable sense of presence did not increase as a result of NF training, neither when looking at the NF-learner group nor in correlation analyses.

### Limitations study 2

As we adapted and transferred the methods from Part 1 of the Study to Part 2 for allowing NF within VR, we had to accept tradeoffs, namely training time and decreased signal-to-noise ratio. The number of training sessions was lower than the average of 7.7 reported in a meta-analysis from Rogala et al. (2016). Comparing further, a recent meta-analysis of studies using NF for treating chronic pain included in the majority at least 12 sessions up to 58 sessions (Hesam-Shariati et al., 2022); however, such a large amount of sessions was not feasible within this project. The choice of sponge-based electrodes on a portable system, the tremendous reduction from 128/64 channels to a subset of 9 channels were tradeoffs that we deemed necessary if aiming at transferring NF technology into VR and broader use of such technology, since the easiness of setting up such a system is a relevant aspect for avoiding non-use (Kübler et al., 2014). As the vision of our project was to train patients with chronic pain such as experienced in fibromyalgia, it was paramount to reduce constraints that were identified in focus groups (conducted specifically for this project) with such patients and health care professionals (unpublished data from Beck, 2023). Electrode preparation time and washing hair after using gel-based electrodes were specifically mentioned as hurdles which confirmed earlier findings (Zickler et al., 2009).

Our training sessions had two different VR scenarios (indoor projector and outdoor fountain), adding complexity for the participants. The duration of a session was short to limit the discomfort associated with the VR HMDs. Training time was reduced from a duration of 7 h30 in Part 1 to 1 h40, which very likely affected learning. Such short training times are suboptimal since learning to regulate a neurophysiological activity requires practice (Birbaumer et al., 1999; Kübler et al., 1999; Lacroix, 1986).

When designing Part 1 of the Study, we were worried that activity in the occipital cortex would result from animated feedback and environment (Händel et al., 2011; Kastner and Ungerleider, 2000) or closing the eyes which may have led to higher alpha in the parietal areas (Barry et al., 2007). Hence, we chose to narrow down the localization of the parietal alpha activity to Pz using CSD spatial patterns. Since the simulated offline reduction to 9 channels showed significant NF ability during Part 1, we used it as spatial filtering also for Part 2. With hindsight, other methods such as simple Laplacian spatial filter, interpolation or average of the parietal channels could have been simple methods to increase signal to noise ratio in the single electrode Pz.

We did not provide participants with a specific mental strategy, which may facilitate skill acquisition as known for regulation of the alpha power over sensorimotor cortices via motor imagery. Our approach was based on the premise that NF learning can be achieved by operant conditioning which does not require a strategy (Sterman and Friar, 1972). Furthermore, we could not find specific strategies recommended, such as for motor imagery, for regulating alpha in the parietal region at channel Pz. Therefore, we assessed the strategies of the participants, but found no conclusive results toward any specific mental imagery.

One may consider the lack of a control group a limitation. However, as we conducted a feasibility Study (Phase 1) it is justified to focus on the practicability of the experimental setup and whether it allows the participants modulating the signal of interest.

A limitation present in both parts of our study is the use of infinite impulse response filters for bandpass filtering, which may introduce a delay between 20 to 100 milliseconds to the real-time bandpassed signal. Since it is known that delaying feedback impedes learning (Belinskaia et al., 2020), finite impulse response filters with mitigation for startup transient may represent a better alternative.

## General discussion

In Part 1 of our study we assessed the feasibility of increasing parietal alpha power via real-time NF to increase the sense of presence in VR. In Part 2 we transferred the training to immersive VR, with a portable and more practical EEG system comprising a reduced number of electrodes.

Using log–log individual regression curves on the performance of single participants allowed us to profile our participants into three groups: learners, fixed performers and inefficient participants. The distribution between these three groups changed substantially between Part 1 and 2. In Part 2 we found no fixed performers. The proportion of participants able to modulate parietal alpha at the end of the training was reduced from 89% in Part 1 to 50% in Part 2, and those were all learners. Results of Part 2 confirm that NF learning is possible within a short training time and with few EEG channels for NF, but longer training may be necessary for some participants and for improving and stabilizing performance (Rogala et al., 2016; Kastner and Ungerleider, 2000). However, the optimal signal specific NF training time is still not known. While VR environments may be more entertaining, they may also distract participants from the main purpose of regulating a specific brain response.

We could not achieve the intended increase of the sense of presence in VR. Sense of presence was high from the beginning and remained stable over time. To address this specific issue, future studies attempting to modulate sense of presence may benefit from alternating between high and low sense presence environments.

## Conclusion

To summarize, (1) Regulating parietal alpha activity was possible after short-term training with few EEG electrodes, but better after long-term training and higher a number of EEG channels that feed into the NF signal. (2) Successful down regulation of parietal alpha power did not affect the sense of presence, at least not if rated high already prior to NF training. (3) Regulation of parietal alpha activity was not linked to frontal–parietal connectivity changes. (4) Enriched and more natural NF (fountain), as compared to classic bar feedback, did not increase performance. (5) No specific strategies to regulate parietal alpha activity were identified. From these results we cautiously conclude that NF learning in a practical VR setup is possible provided substantial training time. Enriched NF may hamper performance due to more sensory input and a more complex NF signal. Decreased parietal alpha power does not lead to an increased sense of presence, at least not if participants already feel highly present at the beginning.

## Data Availability

The raw data supporting the conclusions of this article is openly available on the openneuro platform (Stanford Center for Reproducible Neuroscience). Part 1: 10.18112/openneuro.ds005846.v1.0.0 Part 2: 10.18112/openneuro.ds005878.v1.0.0.

## Footnotes

^1^ https://aspredicted.org/9DG_2ZZ

^2^ https://github.com/chkothe/pylsl

^3^ https://github.com/labstreaminglayer/App-LabRecorder

## Supplementary material

The Supplementary material, including the CRED-NF checklist for this article can be found online at: https://www.frontiersin.org/articles/10.3389/fnins.2025.1476264/full#supplementary-material
